# Facial modeling and measurement based upon homologous topographical features

**DOI:** 10.1371/journal.pone.0304561

**Published:** 2024-05-31

**Authors:** Sawitree Wisetchat, Kent A. Stevens, Stephen R. Frost

**Affiliations:** 1 Department of Anthropology, University of Oregon, Eugene, Oregon, United States of America; 2 Department of Computer and Information Science, University of Oregon, Eugene, Oregon, United States of America; Universidad Nacional de la Plata Facultad de Ciencias Naturales y Museo, ARGENTINA

## Abstract

Measurement of human faces is fundamental to many applications from recognition to genetic phenotyping. While anthropometric landmarks provide a conventional set of homologous measurement points, digital scans are increasingly used for facial measurement, despite the difficulties in establishing their homology. We introduce an alternative basis for facial measurement, which 1) provides a richer information density than discrete point measurements, 2) derives its homology from shared facial topography (ridges, folds, *etc*.), and 3) quantifies local morphological variation following the conventions and practices of anatomical description. A parametric model that permits matching a broad range of facial variation by the adjustment of 71 parameters is demonstrated by modeling a sample of 80 adult human faces. The surface of the parametric model can be adjusted to match each photogrammetric surface mesh generally to within 1 mm, demonstrating a novel and efficient means for facial shape encoding. We examine how well this scheme quantifies facial shape and variation with respect to geographic ancestry and sex. We compare this analysis with a more conventional, landmark-based geometric morphometric (GMM) study with 43 landmarks placed on the same set of scans. Our multivariate statistical analysis using the 71 attribute values separates geographic ancestry groups and sexes with a high degree of reliability, and these results are broadly similar to those from GMM, but with some key differences that we discuss. This approach is compared with conventional, non-parametric methods for the quantification of facial shape, including generality, information density, and the separation of size and shape. Potential uses for phenotypic and dysmorphology studies are also discussed.

## 1. Introduction

### 1.1 Summary

In this paper we describe a new approach to measure human facial shape. Comparative measurements must be homologous, *i*.*e*., comparable across instances. The most broadly-adopted homology is a standardized set of discrete anatomical landmarks (*e*.*g*., [1, 2]). While landmarks commonly serve as the basis for measurements for both anthropometric and geometric morphometric methods, they are sparse and unevenly distributed across the face, and individually carry little shape information. We suggest an alternative homology based upon facial topographic features (*e*.*g*., [3, 4]) used in clinical and anatomical description, allowing us to adopt the associated conventions and terminology of facial features [5, 6]. We also build upon the practices of parametric surface modeling [7–11], adopting their mathematical techniques for representing smooth surface shape and controlling its morphology by discrete control parameters. In our application, the common homologous *topography* of human faces is represented by a continuous surface, the *morphology* of which is then modified using a discrete set of parameters that correspond to elements of anatomical description. Our parameter set corresponds closely to the conventional descriptive lexicon and therefore can be interpreted intuitively and readily visualized, and each carries substantially more shape information than a landmark. As measurements, they are amenable to conventional multivariate statistical and analytical methods.

In this paper we introduce our modeling approach and apply it to a sample of digital scans of 80 adult human faces (composed of equal numbers of males and females of East Asian and European ancestries). Each face is modeled parametrically, resulting in a vector of 71 parameter values that creates a close fit between the surfaces of the model and scan. We evaluate the utility of this method by applying a series of conventional multivariate biometrical methods, and compare the results with those derived by a conventional morphometric analysis of 43 landmarks placed upon the same 80 scans. Similar to conventional GMM, statistical results from our parametric data set can also be visualized in ‘specimen space’.

### 1.2 Background

The human face is fascinating in its complexity and subtlety, exhibiting both systematic variations *and* systematic regularities and constraints. Facial shape is sufficiently constrained as to set expectations for both its norms and ideal proportions, often in the context of beauty and aesthetics. Facial proportions, originally idealized by classical Greeks and then developed as ‘neoclassical canons’ [12–14], were systematically studied with the development of anthropometry. The standardization of craniofacial landmarks [1, 15, 16] permitted the measurement of those ‘norms’ for various groups as well as deviations from those norms [5, 17]. Anthropometric studies have focused largely upon select pairs of landmarks that measure intuitive distances such as the dimensions of the nose and mouth. Ratios of such distances, or ‘indices’, have also been proposed to measure facial proportions and disproportions [18].

While faces tend to vary with ancestry, the individual variation that makes our faces distinctive and recognizable [19] results in statistical variance within a geographic population that is often greater than the mean differences across populations for most anthropometric dimensions [20–22]. It has proven difficult to separate the various contributions towards facial shape and form simply based on the distances between selected anthropometric landmarks, especially given their paucity and uneven spatial distribution across the face. This led to geometric morphometric methods (GMM) to reduce the potential for observer bias and prior expectations in the choice of anthropometric measurements and to better isolate ‘shape’ from size and other aspects of ‘form’ while maintaining spatial relationships [23–27].

In GMM, point measurements are collected, transformed by Generalized Procrustes Analysis (GPA) to factor out orientation, translation, and size, then analyzed to reveal global patterns of covariation, with the final step “graphically visualizing the results of the statistical analyses” [28, 29]. The displacement of Procrustes-aligned landmarks is commonly visualized by heatmaps, difference-vector (‘lollipop’) diagrams, transformation grids, and ‘warps’ [30–32]. However visualized, landmark displacements are to be interpreted relative to other landmarks [23, 29]. Despite theoretical restrictions on their interpretation [31, 33, 34], however, GMM results are often discussed directly in terms of localized changes in the shape of facial features (*e*.*g*., [35, 36]). While intended to analyze shape, the geometric morphometric workflow begins with, and ends with, points—shape *per se* remains implicit in the visualizations used to reveal shape differences.

Another traditional approach, anatomical description, has been standardized since at least the 19^th^ Century [3]. The face is partitioned into regions (*e*.*g*., nose, mouth), and each region is further subdivided into its component anatomical features. Faces are described and compared primarily in terms of the shapes and proportions of its features. The terms used to describe facial features reveal expectations for what would be their normal or representative proportions and shapes, with emphasis placed upon extreme deviations from those expectations (*e*.*g*., a nasal dorsum may be relatively ‘narrow’ or ‘protruding’). This terminology has been standardized, driven by the need to have *“*… *uniform and internationally accepted terms to describe the human phenotype”* intended to facilitate the use of clinical data *“*… *for studies of etiology and pathogenesis*, *epidemiology*, *the isolation of causative gene mutations*, *and for evaluation of interventions”* [5, 6]. This effort includes the features of the cranium and midface [37], nose and philtrum [38], mouth [39], periorbital region [40], and prominent facial creases and folds [4]. Each facial feature is described by a small number of modifiers (adjectives) that are aligned with the conventional anatomical directions (*e*.*g*., mediolateral ‘widths’ and anteroposterior ‘protrusions’). While this lexicon is generally applicable to describing normal structure and morphology, effort has gone towards standardizing the terminology of facial dysmorphologies, which is of particular relevance to the identification of phenotypes. Each property has a presumed normal range of variation, beyond which the feature is regarded as dysmorphic, a classification sometimes based on an objective statistical basis, but more commonly based upon clinical expertise [5].

While anthropometric landmarks are mathematical points, anatomical features are locales. Their boundaries are usually indeterminate (one blends to the next). Nonetheless, by considering the human face as a mosaic of homologous features, anatomical description of the face reduces to their individual descriptions. As discussed next, this homology is based upon surface topography.

#### 1.2.1 Topographical description

Topographic terms such as ‘ridge’, ‘groove’, and ‘fold’ have the same meaning in geology, anatomy, and in common usage. They are intuitive and may reflect how we visually perceive and reason about surfaces and surface curvature [41–43]. Traditional anatomical description is based upon topography, as indicated by anatomical names (*e*.*g*., the supraorbital ridge, alar crease, labiomental sulcus, epicanthal fold).

**Fig 1:**
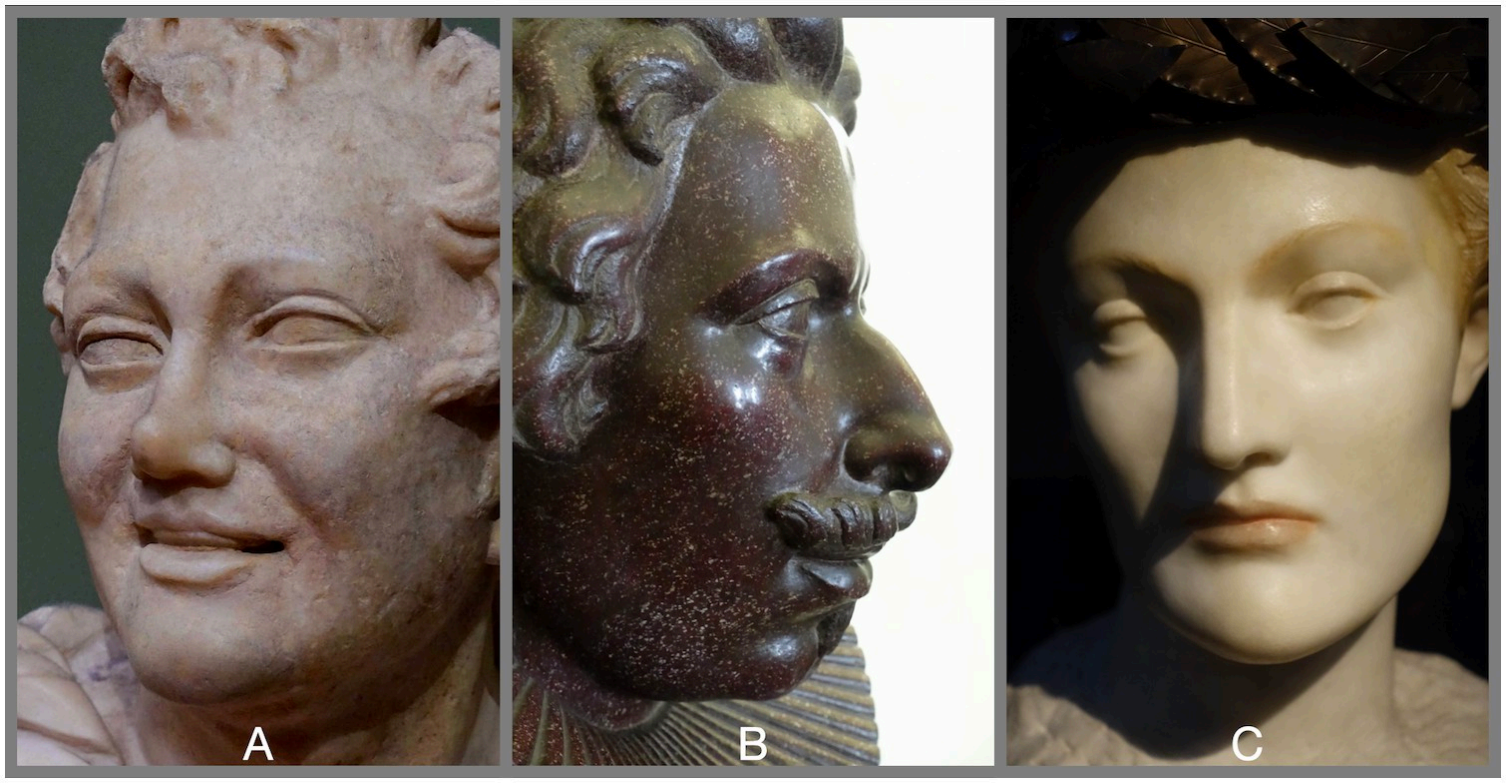
Modeling facial topography. These sculptures demonstrate the homology provided by shared topographic features, and the morphological variations that make individuals distinctive. Each sculpture is a ‘model’—a simplification, an abstraction, that addresses only selected, salient properties of an object at the omission of others. (A) *Head of a Satyr*, Roman, 2^nd^ Century AD, Uffizi Gallery; (B) *Busto di Cosimo II de’ Medici*, Tommaso Fedeli, 1624, Uffizi Gallery; (C) *Futur ou Une jeune femme anglaise*, Fernand Khnopff, 1898, Musee D’Orsay. Photos by Kent A. Stevens.

The topography of the human face is constrained by its underlying anatomy and physiology, which in turn reflects its homologous developmental pathways and shared evolutionary history: *“Your face is the same as everybody has—the two eyes*, *so—’ (marking their places in the air with his thumb) ’nose in the middle*, *mouth under*. *It’s always the same*.*”*[44]. Each region of the face is likewise predictably similar, at least in terms of topography, sharing a common arrangement of anatomical features, or ‘topographic subunits’ [45, 46]. While faces share a common arrangement of topographic features, they vary in the morphology of those features. In comparing the sculptures in Fig 1, for example, the nasal radix is recessed in (A) and prominent in (C), the nasal dorsum protrudes dramatically in (B) yet is straight in (C) and recessed in (A), the alar crease is faint in (C) yet deep in (B), and so forth.

#### 1.2.2 Parametric surface modeling and deformation

A surface is commonly represented by a dense set of three-dimensional point measurements, usually organized into a polygonal mesh, and acquired by various scan techniques [47]. Using heuristics to create an approximate pointwise homology across scans, a statistical analysis of a sample population of face scans can be subjected to principal components analysis to create a parametric ‘face space’ [8]—an extension of the ‘Eigenface’ concept [48, 49] that was originally applied to two-dimensional face images. There has subsequently been a proliferation of applications of ‘morphable models’ for face recognition and face synthesis—see reviews [11, 50]. As these models are usually based upon principal components analysis, their parameters are global and “… do not coincide with attributes that humans would use to describe a face” [50].

**Fig 2:**
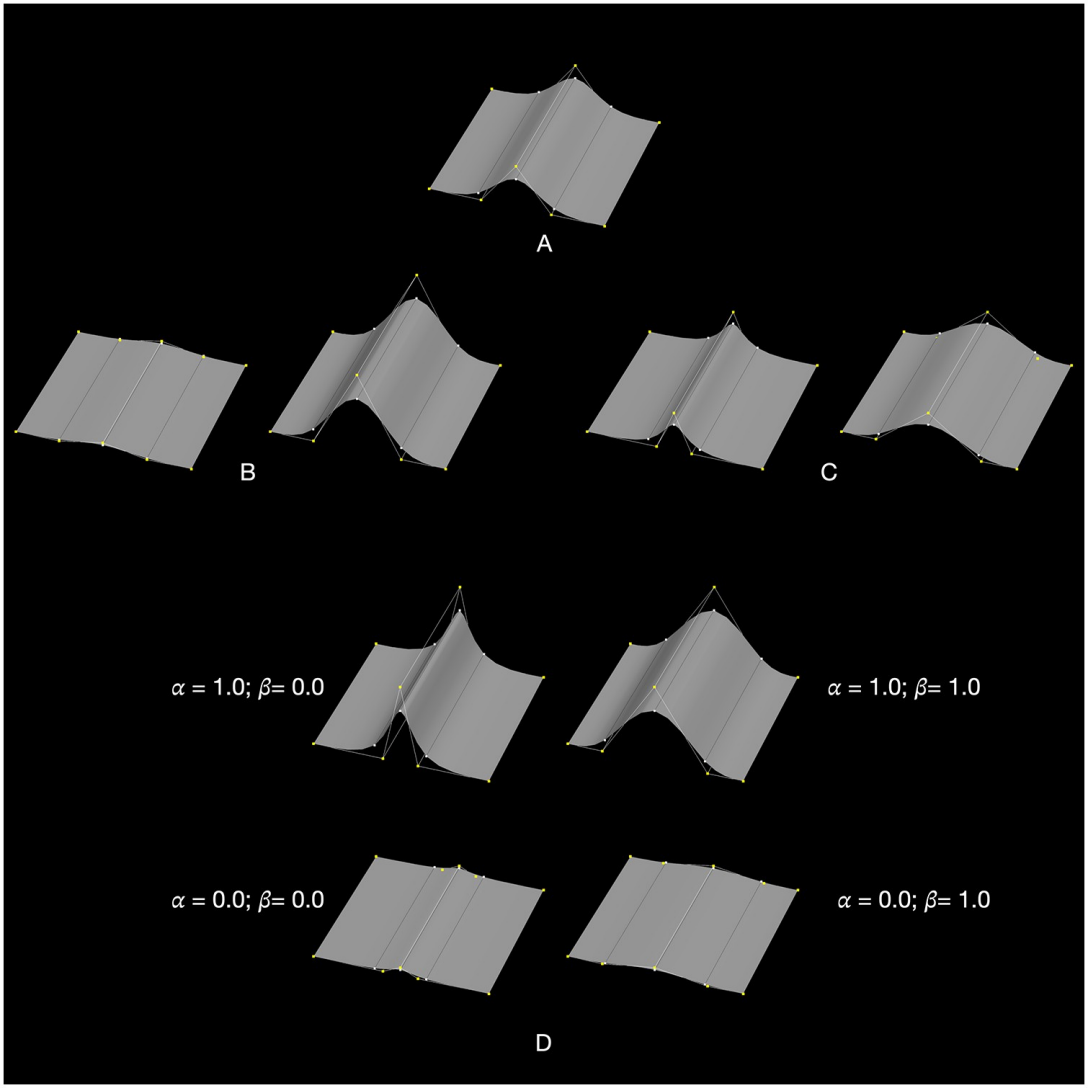
Modeling feature attributes by blendshape deformers. The smooth ridge in (A) is a Catmull-Clark ‘subdivision surface’ [51] created by the recursive subdivision of a simple polygonal mesh of ‘control vertices’. The vertices of the polygonal mesh are shown in yellow. The white vertices on the surface are the interpolated counterparts of the control vertices upon the surface shown in white. The smooth surface is manipulated indirectly by adjusting these control vertices, much as the shape of a Bézier curve is adjusted by shifting its control points. In this way, discrete attributes such as the height and width of the ridge can be implemented by shifting select control vertices. Here, blendshape deformers are used to modify the positions of control vertices. For example, ridge height can be controlled by a deformer that interpolates (with some coefficient *α*) between two homologous meshes that represent two height extremes (B). Likewise, ridge width is modified by a second blendshape deformer that interpolates between the meshes in (C) by some coefficient *β*. The results of the two deformations can then be combined (D) for various *α* and *β*. Since the two deformers create displacements in perpendicular directions, they do not create ‘blendshape interference’ (see text). The position of the ridge could be added as another independent attribute by a third blendshape that shifts all control points associated with the ridge. For further discussion of subdivision surfaces and blendshape deformers, see [51–58].

A very different foundation for surface modeling has been developed in the context of digital animation, which differs in both how surfaces are represented and how they are deformed (‘morphed’). Instead of a dense polygonal mesh, a smooth surface is created by a recursive ‘subdivision surface’ process, wherein a sparse mesh of ‘control vertices’ are progressively subdivided [51]. The subdivision surface can then be deformed by shifting its control vertices. Digital animation and character design use various ‘deformers’ [9, 52–55] to control surface shape, in particular the ‘blendshape deformer’ [52]. Blendshape deformation is used here because it provides a means to precisely quantify the specific shape properties to be associated with individual facial features (*e*.*g*., the protrusion of a ridge, the depth of a sulcus). Blendshape deformation creates a linear blend between two polygons, a ‘base’ B and a ‘target’ T (in our case, both are the control vertices of subdivision surfaces). The base and target must be homologous (*i*.*e*., have the same mesh topology and a one-to-one correspondence between the *i*-th vertex in B and its counterpart in T). The blendshape deformer shifts each vertex B[*i*] a fraction α (0.0 ≤ *α* ≤ 1.0) of the distance to its counterpart T[*i*], *i*.*e*., given B[*i*] = (x_B_, y_B_, z_B_) and T[*i*] = (x_T_, y_T_, z_T_), the deformer results in B[*i*] = (x_B_+*α*(x_T_-x_B_), y_B_+*α*(y_T_ -y_B_), z_B_+*α*(z_T_-z_B_)). Multiple blendshapes can be applied in parallel, wherein the position of each vertex of that mesh is the sum of its original position and the incremental displacements induced by multiple blendshape deformers. The resulting net deformation to the subdivision surface preserves a smooth ‘faired surface’ [56] that blends with the surroundings that are not explicitly deformed. To illustrate, a ridge (Fig 2) was created by a subdivision surface (A). Two blendshape deformers, one based upon a pair of extremes of ridge height (B) and another on extremes of ridge width (C), are applied simultaneously to create combinations of ridge height and width (D).

Blendshapes are often used to animate facial expressions by applying multiple deformations to the same control vertices, however their simultaneous application often results in ‘blendshape interference’ wherein adjusting one interferes with what had just been achieved while adjusting another [10], requiring additional ‘corrective blendshapes’ to counteract undesired artifacts [54, 57, 58]. In the present application, however, blendshapes will be used to model *independent* feature attributes, hence the deformations they create must be linear and additive; care is therefore taken to restrict each blendshape to creating displacements in but one of three orthogonal orientations.

The parametric deformation of a surface, whether achieved by manipulating the weights of eigenvectors or the weights of blendshape deformers, involves a model to be deformed.

Morphable models are often compiled by sampling a population to create a representative surface [59–61]. In the current study a deformable surface represents the common shared topography of human faces while their morphological variations are quantified and represented by blendshape deformation. That is, the deformable surface is implemented by a subdivision surface and each of our facial attributes is implemented by a separate blendshape deformer and quantified by its linear interpolation coefficient. Neither the subdivision surface nor the blendshapes represent population averages.

## 2. The Topographical Face Model

Wisetchat [62] developed the facial modeling system used in this study, referred to here as the Topographical Face Model (TFM). The TFM consists of a digital model of the basic topography of the human face to be ‘morphed’ under parametric control. The topography is represented by a subdivision surface, the morphology is specified by parameters derived from traditional anatomical terminology, and implemented by blendshape deformers. The figures in this paper are rendered images of models created in the TFM. The TFM runs in the 3D environment of Maya^®^ (Autodesk Inc., San Rafael, CA, USA) with the user interface implemented in Python [63]. The TFM implementation is under development and not currently available for distribution.

The TFM creates a full-scale model of a scanned face, without the size normalization performed in conventional GPA, since the absolute scale of adult human faces varies little (face length and width generally vary less than 5% within populations and 20% across populations [15, 17, 21]). The attributes have sufficient range to accommodate this narrow range of absolute size and shape variation in adult human faces. The TFM could also be applied to size-normalized surface scans if desired. When used to model a specific digital scan, the surface of the model is adjusted parametrically to approximate the scan, whereupon the resulting parameter values can then serve as a novel, highly compact, set of facial measurements of the scanned face. Model values are stored in JSON file format and exported to CSV format for analysis. Our study did not address facial asymmetry, therefore we modeled individuals based on the scan data on the left half of the face.

The TFM can operate bidirectionally—to visualize a face given a description, or to create a description given a face. The term ‘model’ refers here to either a vector of attribute values specific to a particular face (or composite of multiple faces), or to the corresponding three-dimensional representation of that face.

### 2.1 Modeled facial features and their attributes

Our model incorporates 36 topographical facial features compiled from the anatomical and clinical lexicon [5, 6, 37–40]. This selection is representative of the shared facial topography, but not comprehensive. We note that a morphometric study by Claes et al. [64] produced a hierarchical decomposition of the face into co-varying ‘segments’ over a range of scales, with 25 of their 32 smallest-scale segments corresponding to the anatomical facial features used here (Table 1).

**Table 1:** Attributes of facial features. Thirty-six facial features are tabulated below, each with associated attributes, for a total of 71 attributes. Widths are mediolateral; lengths and heights are superoinferior; and protrusions and depths are anteroposterior; x, y, and z are positions in these same axes respectively. Throughout this paper, we systematically refer to these anatomical terms by the abbreviations listed here, such as “*FOR_protrusion*” rather than the more cumbersome “anteroposterior protrusion of the forehead”.

Region	Feature (Abbreviation)	Attributes
Forehead	Forehead (*FOR_*)	*protrusion*, *width*
	Supraorbital Ridge (*SOR_*)	*height*, *protrusion*
	Temporalis (*TMP_*)	*width*
	Trichion (*TCH_*)	*protrusion*, *width*
Periorbital	Endocanthion (*ENC_*)	*x*, *y*
	Epicanthal Fold (*ECF_*)	*weight*
	Exocanthion (*EXC_*)	*x*, *y*
	Inferior Palpebral Convexity (*IPC_*)	*weight*
	Interpupillary Distance (*IPD_*)	*x*
	Lateral Palpebral Fossa (*LPF_*)	*depth*
	Medial Palpebral Fossa (*MPF_*)	*depth*
	Palpebral Fissure (*PF_*)	*height*
	Palpebromalar Sulcus (*PMS_*)	*depth*
	Superior Palpebral Convexity (*SPC_*)	*weight*
	Superior Palpebral Sulcus (*SPS_*)	*depth*
	Supratarsal Fold (*STF_*)	*weight*
Mid Face	Cheek (*CHK_*)	*protrusion*, *width*
	Maxilla (*MAX_*)	*protrusion*
	Tragion (*TRG_*)	*x*, *y*, *z*
	Zygion (ZYG_)	*protrusion*, *width*
Nasal	Ala Nasi (*ALA_*)	*drop*, *width*
	Columella (*COL_*)	*drop*, *show*, *width*
	Nasal Dorsum (*DSM_*)	*length*, *protrusion*, *width*
	Nasal Sidewall (*NSW_*)	*slope*
	Nasal Radix (*RAD_*)	*height*, *protrusion*, *width*
	Supra-Alar Crease (*SAC_*)	*weight*
	Supratip Break (*STB_*)	*weight*, *width*
	Nasal Tip (*TIP_*)	*inclination*, *protrusion*, *width*
Perioral	Cheilion (*CH_*)	*height*, *protrusion*, *width*
	Inferior Labium (*IL_*)	*curve*, *fullness*, *protrusion*, *thickness*
	Philtrum (*PHL_*)	*depth*, *length*, *protrusion*, *width*
	Superior Labium (*SL_*)	*convexity*, *curve*, *fullness*, *protrusion*, *thickness*
Lower Face	Chin (GNA_)	*length*, *protrusion*, *width*
	Gonion (*GON_*)	*height*, *protrusion*, *width*
	Labiomental Sulcus (*LMS_*)	*weight*
	Mandible (*MDB_*)	*width*

The adjectives used in medical and clinical descriptions describe the dimensions and shapes of each feature relative to anatomical directions, such as the superoinferior length and mediolateral width of the philtrum (*PHL_length*, and *PHL_width*). Each descriptor is quantified over a normalized range from 0.0 to 1.0 (*e*.*g*., from ‘short’ to ‘long’, or ‘narrow’ to ‘wide’) sufficient to cover expected variation; it is also possible to linearly extrapolate beyond that range. Attribute values will act like interval data [65]. There is no expectation for a mean value (*e*.*g*., a ‘normal length’), and an attribute value of 0.5 has no statistical significance—it is simply halfway between those two extremes. The ‘meaning’ of an attribute value is three-dimensional and implemented by an associated blendshape deformer which creates a measured deformation to a corresponding area of the surface, as discussed next.

### 2.2 Representing facial attributes as three-dimensional shapes

Our goal is to encode facial shape by a fixed set of parameters which correspond to the adjectives used for anatomical description, with the intended product a set of parameter values that can serve as facial measurements. In accordance with conventional practice, we follow a reductionist approach towards modeling human faces, beginning with decomposition of the whole into its anatomical features, each of which is described in terms of a few attributes—reducing facial description to the sum of the descriptions of its features. As is common practice, we represent the face as a surface that is subject to deformations. The novel aspect of our study is the effort to create a direct, one-to-one mapping between a specific facial morphology and a set of attribute values. If successful, the vector of attribute values would encode a useful approximation to the shape of a specific face.

Our approach can encode any shape variation that can be defined by linear interpolation between a pair of extreme shapes, not merely dimensional aspects of facial features. The epicanthal fold, for example, can be quantified by the attribute *ECF_weight* (which varies from 0.0 if absent to 1.0 if very pronounced) and implemented by an associated blendshape that varies the extent to which the fold overlays the endocanthus and the degree of arcuate shape of the fold.

The facial surface topography was modeled by a subdivision surface with an underlying mesh of 175 control vertices for one side of the face (as it is symmetrical for this study)—providing sufficient complexity to define the 36 facial features in Table 1 and to allow each feature to be deformed according to the 71 attributes in Table 1. The associated 71 blendshape deformers were designed to provide sufficient range of shape variation without blendshape inference.

Anatomically, facial features are conventionally described locally, without reference to their absolute placement on the face, wherein the overall dimensions of the face derive from the summation of the dimensions of multiple features. The TFM model therefore requires a fixed point of reference, or origin, relative to which the features of the face are distributed. A right-handed Cartesian coordinate system is aligned with the anatomical directions (x, y, and z aligned with mediolateral, superoinferior, and anteroposterior, respectively). Since no soft-tissue landmark would serve as a fixed three-dimensional origin for the model, we chose the midpoint between the apices of the left and right corneas to serve as the origin. The direction of gaze is horizontal down the z axis (similar to that of Zhurov et al. [66]), the y axis is vertical, and the model is oriented in conventional ‘natural head position’ [67, 68]. Because features are described locally, without reference to their absolute positions, attributes of more proximal features influence the positions of more distal features. This necessitates that during the modeling process attribute values must be determined in a proximal to distal order.

**Fig 3:**
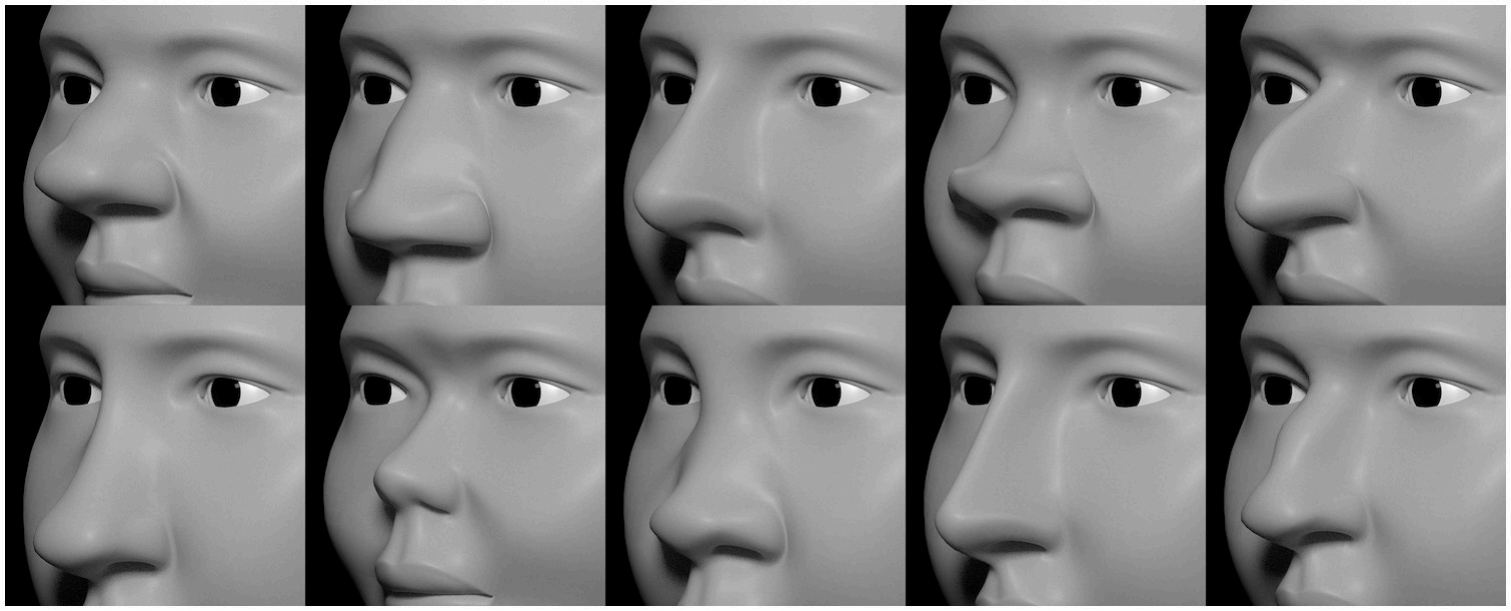
Examples of nose variation created by combinations of 18 nose attributes. Each attribute produces a local deformation restricted to one of three orthogonal orientations. In combination they create a large space of linear combinations of those nose shapes.

To illustrate our approach, consider the modeling of the nose. The topography of the nose is a patchwork of eight adjoining topographic features (the saddle-shaped radix, the ridge-like dorsum, the convex nasal tip, etc.) and a total of 18 attributes (Table 1). Various combinations of these attributes are shown in Fig 3. Note that the superoinferior length of the nose determines the position of the philtrum and other features inferior to it (Fig 3 and S1 Fig).

Additivity also arises anteroposteriorly. The profile of the mid and lower face (as seen in lateral aspect) is traditionally described in terms of maxillary (alveolar) prognathism [69]. This is modeled in the TFM by the attribute *MAX_protrusion*, which shifts entire areas of the mid and lower face anteroposteriorly. The overall protrusion of the tip of the nose (relative to the origin) would be the additive combination of *TIP_protrusion* and the underlying shift due to *MAX_protrusion* (compare S1E and S1H Fig).

While superoinferior lengths and anteroposterior protrusions are modeled by the summation of incremental shifts, the features on the side of the face (those in the vicinity of the tragion, zygion, gonion, *etc*.) are relatively isolated from the features that lie along the sagittal plane (principally those of the nose, eyes, and mouth). Variations in the widths of the medial features (*e*.*g*., *ALA_width*, *CH_width*) do not propagate shifts to more lateral features. Instead, medial and lateral features vary independently in width by displacements relative to the sagittal plane, and smoothly blended across the liminal areas of the cheek and jaw (S2 Fig).

**Fig 4:**
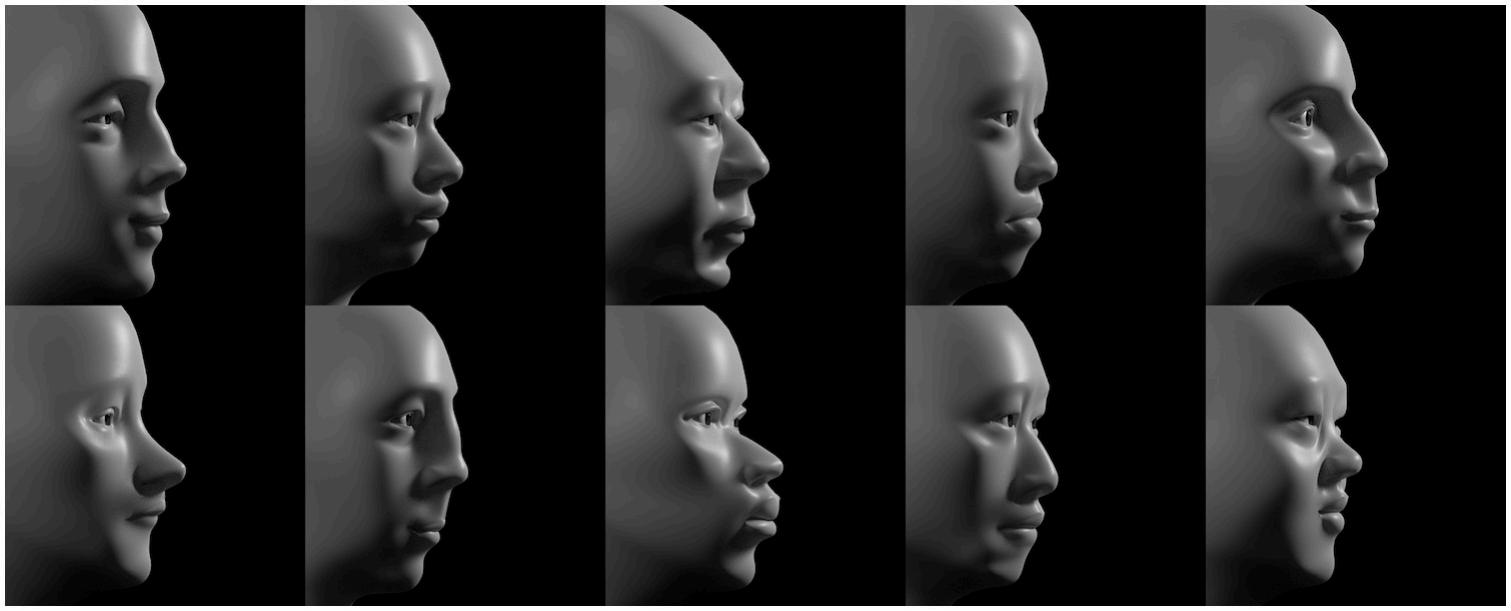
A parametric model with 71 attributes permits a large ‘face space’. To appreciate the space of possible faces, if each attribute were very conservatively assumed to support only four perceptually-distinct values (*i*.*e*., a just noticeable difference of roughly 0.25 in the normalized range from 0.0 to 1.0), the model would permit 4^71^ (*i*.*e*., 10^42^) combinations. See also S3 Movie.

Each attribute corresponds to an independent deformation; the 18 nasal attributes are shown as individual animations in S1 Movie and the 14 periorbital attributes are shown individually in S2 Movie. The overall shape of a face results from the simultaneous application of all 71 deformations; the three-dimensional position of each control point is the algebraic sum of all deformations that affect that point. Fig 4 shows ten examples of a huge space of distinct facial morphologies that can be encoded by the 71 attributes.

The process of modeling a particular scan (S4 Fig) involves the manual adjustment of attributes in a proximal-to-distal order. First, the digital scan is superimposed at the origin in the same placement and orientation as the model (S3 Fig), then the interpupillary separation of the model *IPD_x* is adjusted to superimpose the eyes of the model and those of the scan. Next, the overall width of the head at the tragion (Fig 6 landmark 21) is matched by adjusting *TRG_x*, following which the forehead features can be modeled. Next, *DSM_length* and *MAX_protrusion* are adjusted to match the model and scan in the immediate vicinity of the subalare (Fig 6, landmark 23), whereupon the features of the midface, periorbital, and nasal regions can be modeled. Then, by proceeding inferiorly from the nose, the perioral features are matched followed by those of the lower face.

It is worth noting what is *not* modeled by this approach: The face is not spatially partitioned into discrete regions, neither are regions subdivided into separate facial features. Only the *attributes* themselves have any concrete realization in the TFM, by how they create local deformations in the model. The TFM builds different faces entirely by differential translations of local surface patches.

While the TFM attributes have a unified three-dimensional representation scheme, their values are incommensurate—some are dimensional (*e*.*g*., *DSM_length*), others positional (*e*.*g*., *ECF_x*) and yet others capture the presence of folds or creases (*e*.*g*., *STF_weight*). Consequently, in multivariate statistical analyses of TFM data, we use methods based on correlations rather than covariances (*e*.*g*., in PCA). The advantage is that the TFM data can reveal correlations and other statistical trends directly in terms of these individual attributes.

## 3. Materials and methods

### 3.1 Dataset

We applied the TFM to a set of 80 anonymized stereophotogrammetric scans provided by Prof. L. DeBruine, Department of Psychology and Neuroscience at the University of Glasgow. Prof. DeBruine, as External Supervisor for SW’s dissertation committee, provided SW with data for her dissertation research [62] that had been compiled from a pool of registered experimental subjects governed by the University of Glasgow Human subjects ethics guidelines; this study derives from that data. We have preserved the subject’s facial anonymity and present only composite (averaged) scan images of a minimum of 20 individuals.

The sample consisted of only two ancestry groups: forty individuals who self-identified as having eastern Asian ancestry (EAS) and forty of European ancestry (EUR), each with equal numbers of self-identified females and males. Given the limited sample size, this study is intended only as a demonstration of our approach.

**Fig 5:**
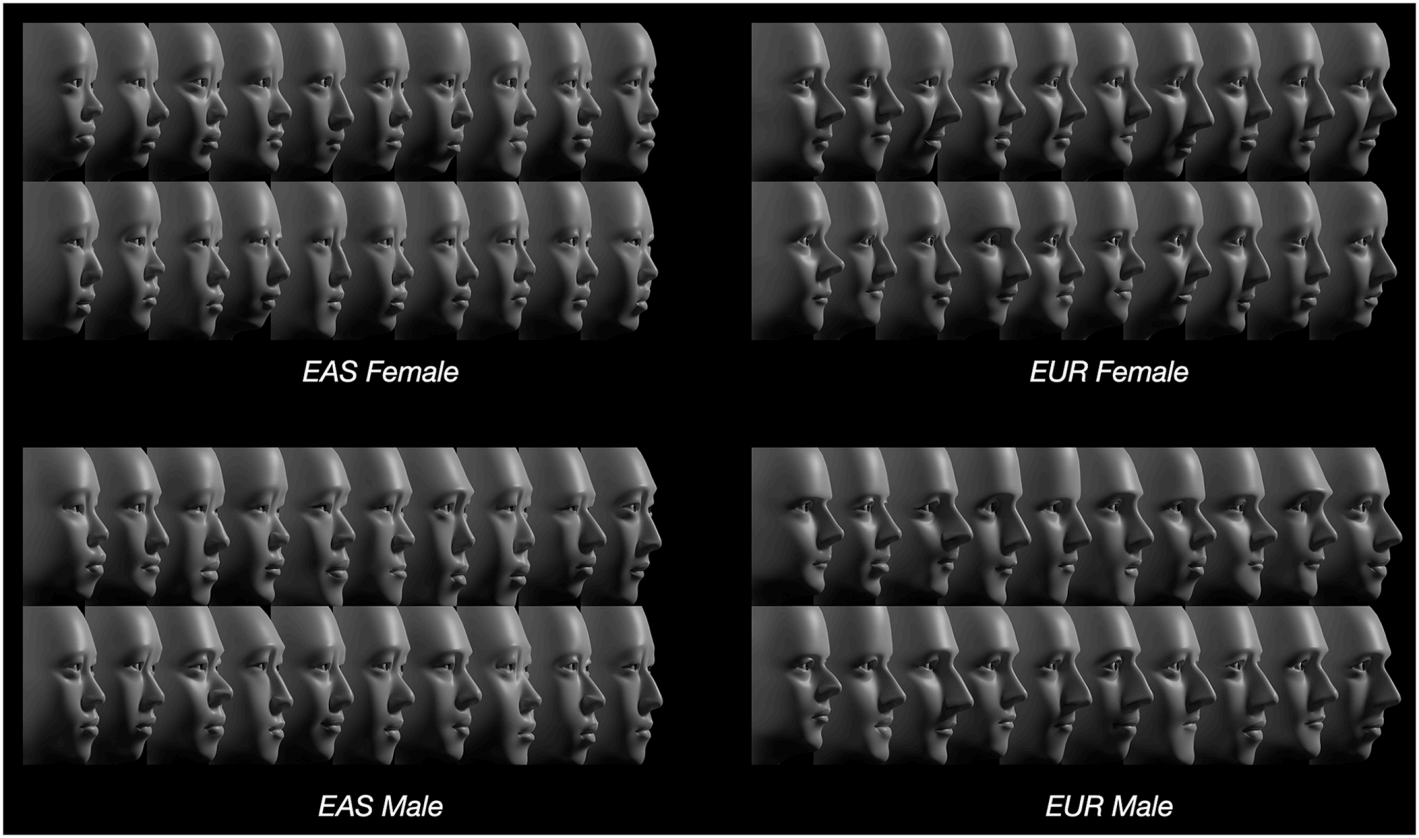
Models of 80 individuals. Each face was modeled parametrically using the TFM to create a close approximation of its corresponding photogrammetric scan (see also S4 Movie).

**Fig 6:**
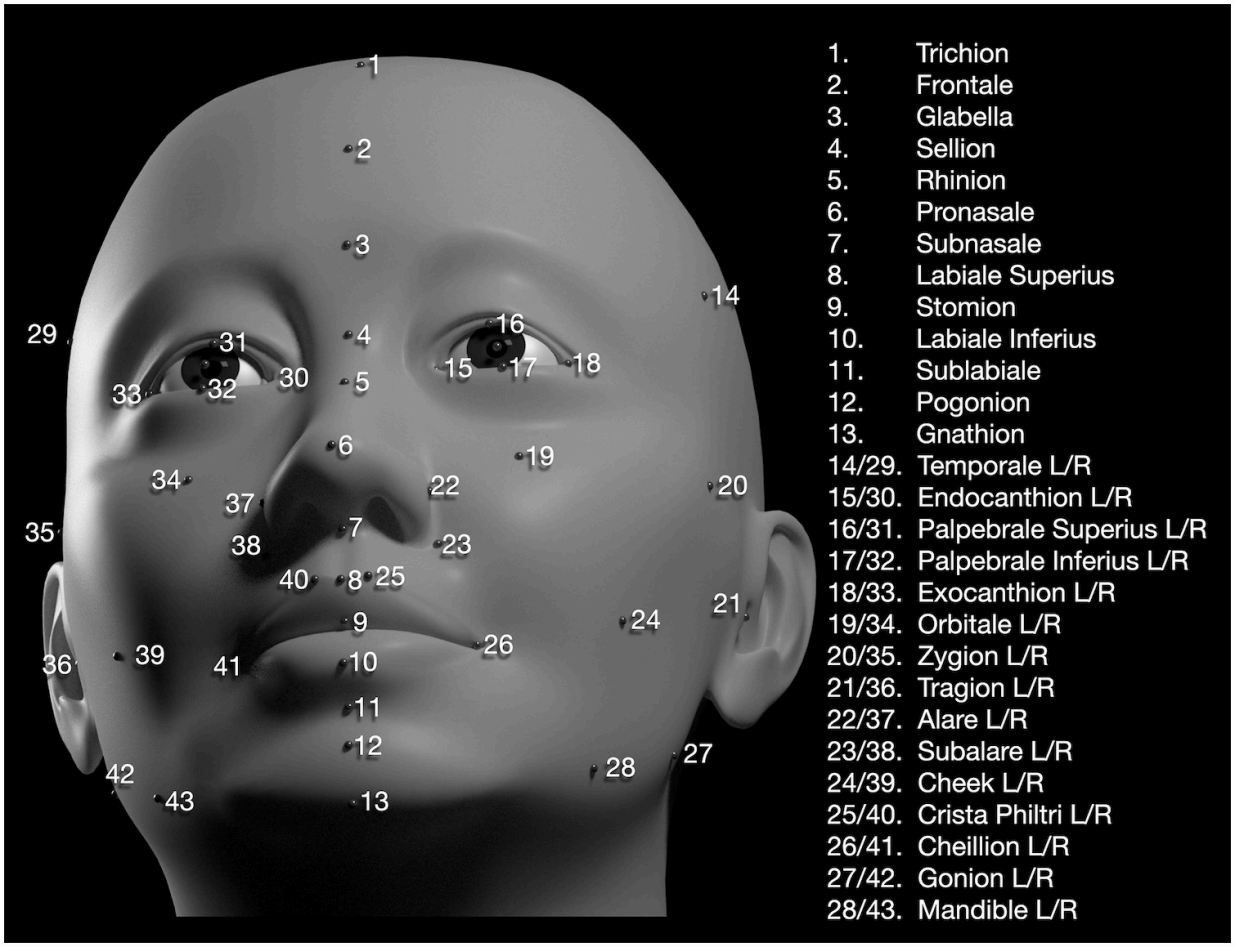
Anthropometric landmarks. Each digital scan was landmarked with 43 conventional landmarks, which includes some new landmarks (2, 14/29, 23/38, 24/39, and 28/43) that provide coverage across areas that are modeled in the TFM. This face is a rendered TFM model.

In addition to the photogrammetric scans of the 80 individuals, four composite scans were included, each the average of 20 individuals. Only these averaged scans are shown in this study to preserve anonymity [70]. The scans were generated using the Di3D^™^ stereophotogrammetry surface imaging system [71], with delineation using MorphAnalyzer 2.4.0 [72]; each consisted of roughly 90,000 vertices. The 80 digital scans were modeled (by SW and KS; Fig 5 and S3 Fig) and landmarked (by SF; Fig 6); all data provided in S1 File.

### 3.2 Evaluation of the method

This paper has two primary goals: 1) demonstrating that facial morphology can be decomposed into a set of linearly-independent shape attributes that summate to reconstruct an overall facial surface, and 2) showing that the attributes can act as the basis for shape measurement methodology for conventional morphometrics.

#### 3.2.1 Measuring and visualizing model accuracy

To assess how closely our models corresponded to the original scan data, we calculated the separation between the modeled surface and the surface of the scan at points across the model. To compare the model (a subdivision surface) with a scan (a polygonal mesh), a polygonal approximation of the subdivision surface is created, and those vertices on the left side of the face serve as a set of 2,096 sample points across the model. The perpendicular separation between the model and the scan is computed by constructing the line segment from each sample point to the nearest scan vertex, then projecting that line segment onto the unit surface normal of the model at that point. The perpendicular separation, measured in millimeters, is then ‘heatmapped’ across the model to visualize the quality of the fit of the model to the data. We use a variant of a common heatmap technique which draws attention to areas of greater disparity between the two surfaces (S4 Fig).

While perpendicular separation is used to compare a model and a scan, a different approach is possible when comparing models. Since all TFM models share a common control polyhedron, the 2,096 sample points are homologous across models, and permit visualizing both the magnitude and the orientation of the deformation at those points.

A variety of visualization techniques are commonly used to compare two surfaces, such as displacement vectors to indicate orientation and magnitude of displacement between homologous points [32], and simple heatmaps to visualize scalar magnitude information alone (*e*.*g*., perpendicular separation). We separate the visualization of magnitude and orientation, using a variant on the standard heatmap to draw attention to areas of greater displacement magnitude (S4 Fig). To visualize the orientation in which this displacement occurs, we use three color channels to encode the three orthogonal orientations (S5 Fig). This technique is used in S6 Fig to show the orientation and the spatial extent of the displacement associated with each of the 71 attributes. Some attributes are very localized such as those in the periorbital region, but a few (*e*.*g*., *DSM_length*, *PHL_length*, and *MAX_protrusion*) shift large areas of the face.

#### 3.2.2 Suitability for morphometric analysis

As a basis for comparison, we performed a non-parametric analysis using the Procrustes method (*e*.*g*., [23, 73, 74]). We digitized 43 standard facial landmarks with Landmark Editor [75] using the same 80 facial scans that were modeled in the TFM. Approximately half of the landmarks follow the protocol of Paternoster et al. [76] from their analysis of correlated genetic information and facial shape. To these we added additional landmarks to more closely approximate the coverage provided by the TFM attributes. Generalized Procrustes superimposition was then completed in MorphoJ [77] and applied to the 80 landmark configurations (*i*.*e*., each set of landmarks for an individual) to remove variation due to absolute scale, position, and orientation; with residuals projected into a Euclidean tangent space making them appropriate for subsequent statistical analyses [28].

The TFM encodes the morphological variation in an individual facial scan as a succinct set of 71 attributes, which can be used directly as variables in conventional multivariate statistical approaches (*e*.*g*., [78, 79]). To this end, we assessed how well these attribute values were able to distinguish subgroups and to characterize patterns of shape variation for the 80 individuals in our dataset.

To quantify absolute scale in the TFM dataset, the attribute *meanVertexRadius* was computed based upon the mean radius of the vertices of the associated photogrammetric scan relative to the mesh centroid. In the Procrustes analysis we used centroid size (the square root of the sum of the squared distances of the 43 landmarks from their common centroid) to measure scale, as is standard in Procrustes analysis [23, 80].

The same set of analytical approaches were used for our two data sets (the 71 TFM attributes and the 129 superimposed Procrustes residuals). Principal Components Analysis (PCA) was used as a data reduction, ordination, and exploration technique for both TFM attribute scores and Procrustes residuals. PCA is a rigid rotation of the data that converts the original variables into a new set of axes (PCs) of full rank which are linear combinations of the originals, independent, ranked by their variance, and have arbitrary positive and negative direction [81]. PCA is a common tool for assessing overall patterns of variation in multivariate data sets [78]. We used the correlation matrix for the PCA of TFM attributes because they are not all in the same units; the PCA of the Procrustes data was based on the variance-covariance matrix as they are all in the same units.

Multivariate analysis of variance (MANOVA) was used to test for differences in mean shape between ancestry groups (*i*.*e*., Eastern Asian and European) and biological sexes (female and male). Because the Procrustes data have more variables (129 coordinates) than there are observations in our data set (n = 80), and likewise the 71 TFM attributes are close to the number of observations, the scores for the first 10 Principal Components from the corresponding PCAs were used in both analyses instead. These represent more than 69% and 55% of the total variance respectively. We tested for multivariate differences in shape using Wilks’s Lambda. Where multivariate differences were significant, specific attributes that differed between groups were identified based on univariate tests with a Bonferroni correction applied (p = 0.05 / 71 attributes = 0.0007). For the TFM data, this produces statistical results that are readily interpreted as conventional anatomical descriptions.

Two-way ANOVAs were used to test for differences in facial size among ancestry groups and sexes using the natural log of centroid size for the Procrustes data and *meanVertexRadius* for and TFM data. Allometry, or change in shape associated with size, is often an important component of shape variation in biological data (*e*.*g*., [82]). Therefore, multivariate analysis of covariance was used to assess the association of facial shape with size [73]. For the Procrustes data, the 129 GPA coordinates were the dependent variables and log transformed centroid size the independent. For the TFM, 71 attributes were treated as dependent variables and *meanVertexRadius* as the independent variable.

Correlations between patterns of shape difference explained by ancestry group, sex, and size (*i*.*e*., *MeanVertexRadius* for TFM and natural log transformed centroid size for Procrustes data) were calculated as the inner dot product of the respective vectors.

Shape changes associated with group means, PCA axes, and regression results were visualized for both datasets. For the landmark data, we computed the vector of 129 Procrustes-aligned coordinates for the grand mean of the 80 individual models. To this vector of grand means, we added and subtracted vectors of coefficients from multivariate linear regression on ancestry, sex, and size. These displaced landmarks were used to warp an exemplar surface using a thin-plate spline [83] to visualize those changes on the original scan data in Landmark editor. We used an analogous approach for the TFM dataset by adding and subtracting vectors of regression coefficients to the grand means of the 71 attributes. We then used these values to produce new TFM models illustrating those specific aspects of shape difference relative to their mean.

## 4. Results

### 4.1 Model accuracy

The dataset of photogrammetric scans provided for this study consisted of scans of 80 individuals, 20 per group. The models are shown in S7 Fig, each accompanied by a heatmap showing the fit between the model and its associated scan; the photogrammetric scans of the individuals are not shown for anonymity. The fit between each model and associated scan was measured by computing the perpendicular separation at each of 2,096 homologous sample points across the surface of each TFM model (Section 3.2.1).

**Fig 7:**
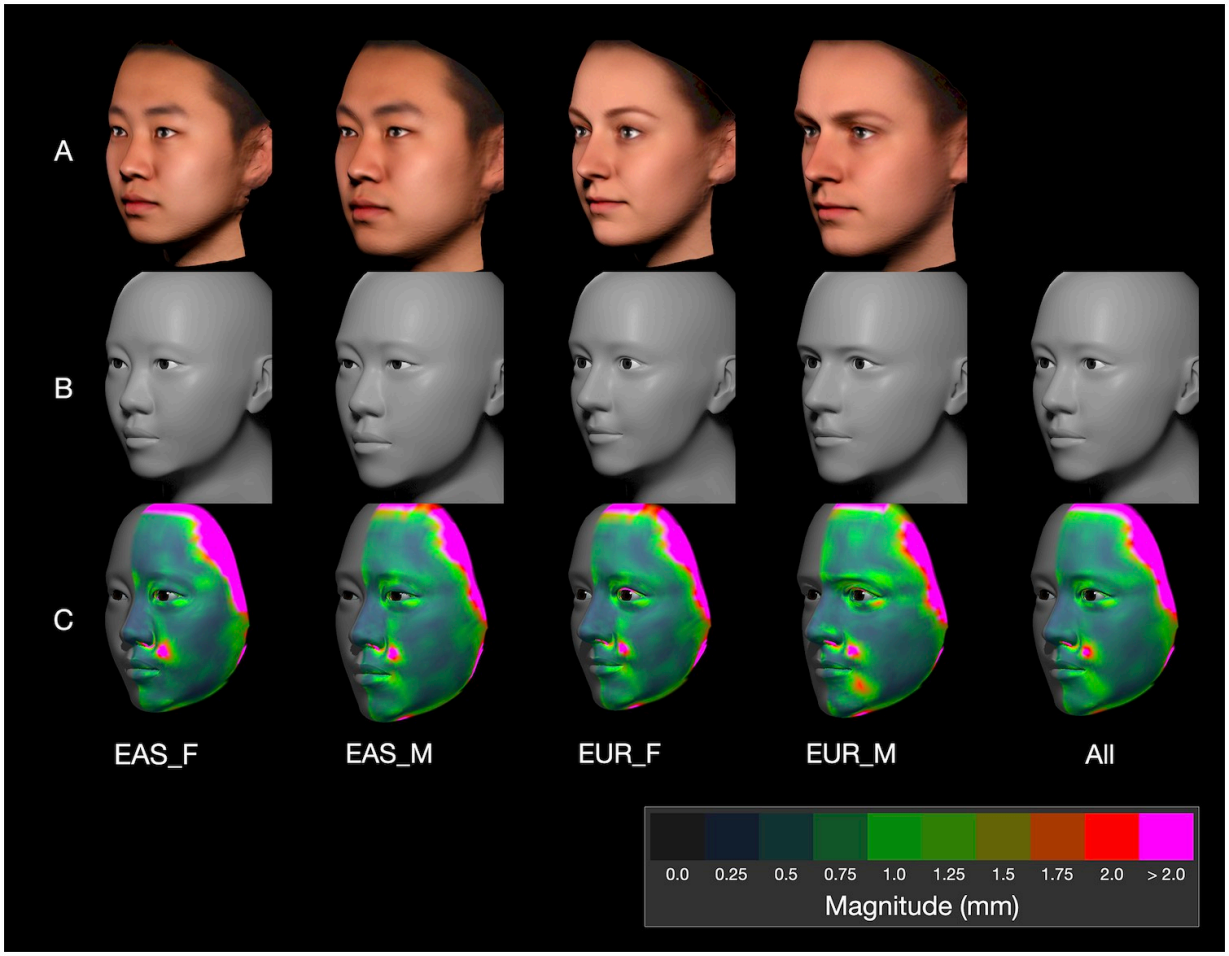
Evaluation of mean fit between model and scan. The photogrammetric averages for each group of 20 individuals are shown in (A) for reference. The models in (B) are the corresponding computed averages of the individual models in each group. The heatmaps in (C) show the mean disparity between each model and its corresponding digital scan. The rightmost model in (B) and its heatmap in (C) are averages based on all 80 models. Table 2 shows that more than 80% of the sample points across the models were separated by less than 1 mm.

This study was provided with four additional composite scans that represent the ‘averaged face’ scan for each group (Fig 7A). A corresponding averaged TFM model was computed (wherein each attribute was the computed mean of the corresponding attribute values for the 20 models for each group). These models are shown in Fig 7B, along with a grand average of all 80 models on the far right. To measure the average fit between model and scan, the mean fit at each of the 2,096 sample points was computed for each group of 20 models and for all 80 models and heatmapped (Fig 7C).

**Table 2:** Percentage of sample points of the model within a given distance of the original scan (millimeters).

	0.0 mm	0.25 mm	0.5 mm	0.75 mm	1.0 mm	1.25 mm	1.5 mm	1.75 mm	2.0 mm
**EUR_F**	16.0%	42.0%	61.0%	74.0%	82.0%	87.0%	90.0%	92.0%	94.0%
**EUR_M**	16.0%	43.0%	62.0%	75.0%	83.0%	88.0%	92.0%	94.0%	96.0%
**EAS_F**	18.0%	46.0%	64.0%	76.0%	83.0%	88.0%	91.0%	93.0%	95.0%
**EAS_M**	16.0%	43.0%	62.0%	74.0%	82.0%	87.0%	90.0%	92.0%	94.0%
**Mean**	16.5%	43.5%	62.3%	74.8%	82.5%	87.5%	90.8%	92.8%	94.8%

### 4.2 Morphometric analysis using parametric and Procrustes data

#### 4.2.1 Principal components analyses

The first ten PCs of the Procrustes Landmark data account for over 69% of their variance, and the first ten PCs of the TFM attribute values account for over 55% of the variation in the data (Table 3).

**Table 3:** The first ten eigenvalues for PCAs of the Procrustes and TFM analyses.

PC	Procrustes Eigenvalue	Procrustes % Variance	TFM Eigenvalue	TFM % Variance
1	0.00156538	22.4%	10.83	15.3%
2	0.00075171	10.8%	6.487	9.14%
3	0.00049509	7.09%	3.837	5.40%
4	0.00042257	6.05%	3.525	4.97%
5	0.00036685	5.25%	2.965	4.18%
6	0.00028553	4.09%	2.638	3.72%
7	0.00027001	3.87%	2.495	3.51%
8	0.00024425	3.50%	2.304	3.25%
9	0.00022360	3.20%	2.187	3.08%
10	0.00020753	2.97%	2.043	2.88%

For the Procrustes data, principal component 1 accounts for 22% of total variance and has EUR at the negative end and EAS at the positive, with some overlap near the origin. Furthermore, within each ancestry group, females are generally more positive, with some overlap within EAS and significant overlap within EUR. In terms of shape differences, Procrustes PC1 indicates a more projecting nose and brow on a narrower mid-face at the negative end and a less projecting nose and brow and wider face at the positive. Procrustes PC2 accounts for about 11% of the total variance in the sample with a relatively narrow and tall face at the negative end and a shorter, wider face at the positive. Unlike Procrustes PC1, PC2 does not effectively separate any of the *a priori* groups. Procrustes PC3 (7% of the total variance) partially separates males and females within EAS, but not EUR. In shape it represents the relative proportion and protrusion of the forehead compared to the lower face.

**Fig 8:**
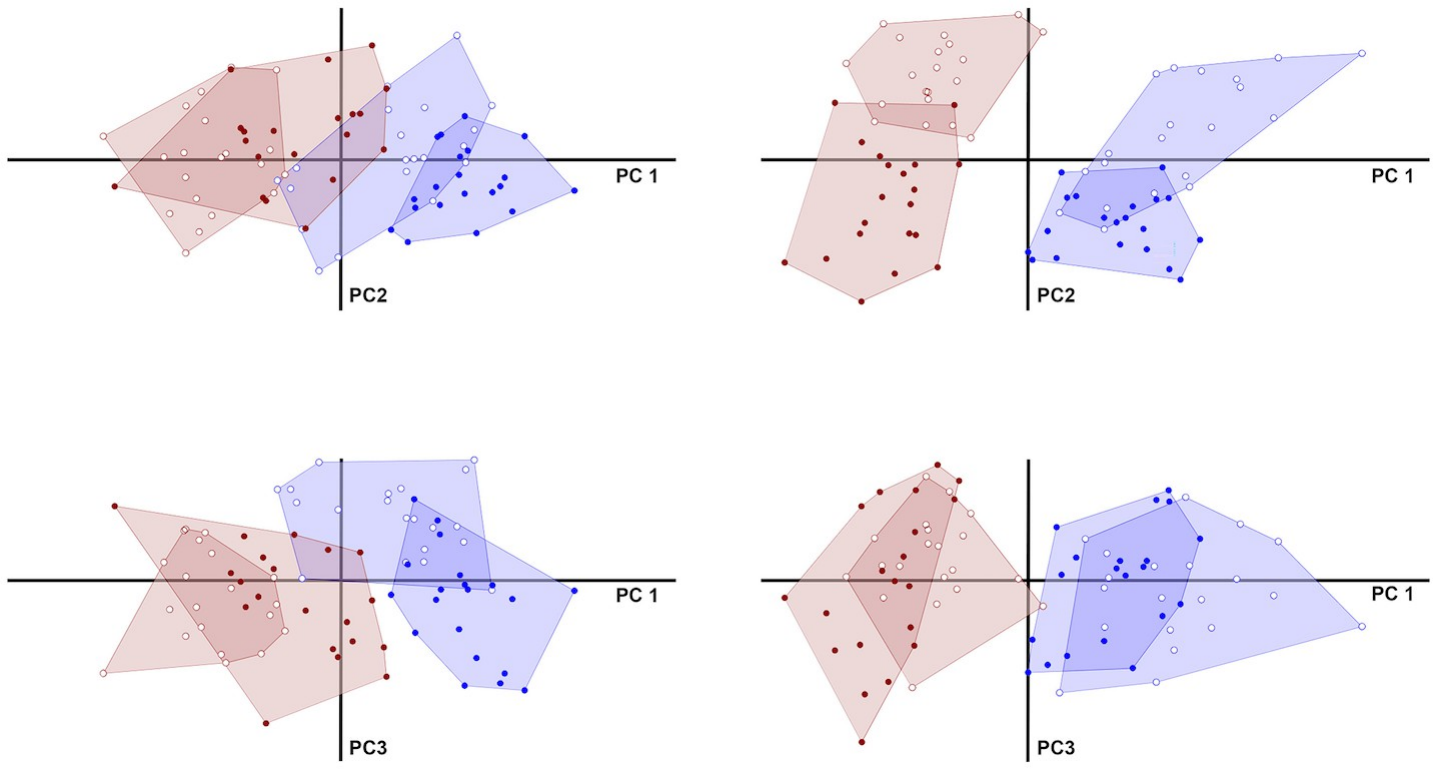
Bivariate plots comparing principal component scores for the Procrustes and TFM datasets. Scores from Procrustes (left) and TFM (right) data are shown for principal components 1 and 2 (top row), and 1 and 3 (bottom row). PCA of the Procrustes data segregates ancestry groups along PC1 fairly well. The sexes appear best differentiated along PC3, but less clearly than along TFM PC 2. For the TFM, PCs 1 and 2 essentially distinguish the two geographic ancestry groups and the two sexes, respectively, whereas all groups largely overlap in PC3. Open/closed circles = Female/Male; red/blue = EUR/EAS.

For the TFM data, the first two PCs clearly separate individuals by *a priori* ancestry and sex groups (Fig 8). The first principal component for the TFM accounts for approximately 15% of the variation in our dataset and largely separates individuals by ancestry group with little overlap, EUR being at the negative end and EAS at the positive. The sexes broadly overlap in their TFM PC1 scores, but with females shifted slightly towards the negative end. The facial attributes that load most strongly on TFM PC1 include epicanthal fold weight (*ECF_weight*), the mouth (*CH_protrusion*, *IL_protrusion*, *SL_thickness*, and *SL_protrusion*), and the nose (*ALA_width*, *RAD_protrusion*, *DSM_protrusion*, and *TIP_protrusion*). TFM PC2 accounts for approximately 9% of overall variation and broadly separates individuals by sex, albeit with considerably more overlap, females having lower values compared to males (Fig 8). There is substantially less overlap between the sexes within each geographic ancestry group. The attributes that load most strongly on TFM PC2 are *SOR_protrusion*, *GON_height*, *IPD_x*, *MAX_protrusion*, and *TIP_protrusion*.

#### 4.2.2 Multivariate analysis of variance

For the Procrustes data, multivariate analysis of variance of the scores from PCs 1–10 differed by geographic ancestry group (*p* < 0.0001) and sex (*p* < 0.0001) with a significant interaction (*p* < 0.0001). Sexes were different in facial shape within EAS (*p* < 0.0001) and EUR (*p* = 0.0017); as were ancestry groups within females (*p* < 0.0001) and males (*p* < 0.0001). Size as measured by the natural log of centroid size differed by ancestry group (*p* < 0.0001) and sex (*p* < 0.0001) with no significant interaction (*p* = 0.0802).

For the TFM data, multivariate analysis of variance of the scores from PCs 1–10 differed by both geographic ancestry group (*p* < 0.0001) and sex (*p* < 0.0001). There was no interaction between ancestry and sex (*p* = 0.0683). Table 4 gives the 20 attributes that differ significantly (*p* = 0.0007) between ancestry groups, and Table 5 the 12 significantly different attributes (*p* = 0.0007) between sexes. Size as measured by *meanVertexRadius* differed by ancestry group (*p* = 0.0006) and sex (*p* < 0.0001) with no significant interaction (*p* = 0.3512).

**Table 4:** Attribute comparison of 40 European versus 40 East Asian models (pooled sex). Geographic ancestry groups were found to differ in mean shape based on Wilks’s Lambda. Those individual attributes that were significantly different based on univariate t-tests with Bonferroni correction are listed. Attributes are sorted in order of decreasing difference between means (Δ) as quantified in terms of standard deviations.

Attribute	Region	Description	EAS *X*	EUR *X*	Δ
*ECF_weight*	Periorbital	epicanthal fold	0.793	0.000	1.830
*DSM_protrusion*	Nasal	dorsum protrusion	0.184	0.622	1.629
*RAD_protrusion*	Nasal	radix protrusion	0.225	0.591	1.592
*TIP_protrusion*	Nasal	nasal tip protrusion	0.127	0.447	1.567
*ALA_width*	Nasal	alar width	0.314	0.129	1.300
*MAX_protrusion*	Mid Face	maxillary protrusion	0.164	0.373	1.248
*SL_thickness*	Perioral	superior labium thickness	0.548	0.243	1.209
*SOR_protrusion*	Forehead	supraorbital ridge protrusion	0.217	0.463	1.197
*FOR_protrusion*	Forehead	forehead protrusion	0.499	0.678	1.126
*CH_protrusion*	Perioral	cheilion protrusion	0.707	0.421	1.109
*ZYG_width*	Mid Face	zygion width	0.467	0.215	1.085
*SPS_weight*	Periorbital	suprapalpebral sulcus	0.463	0.171	1.048
*PTF_depth*	Mid Face	palpebrotemporal fossa	0.574	0.769	1.000
*RAD_height*	Nasal	radix height	0.353	0.623	0.998
*SL_protrusion*	Perioral	superior labium protrusion	0.390	0.225	0.984
*IL_protrusion*	Perioral	inferior labium protrusion	0.649	0.438	0.919
*ENC_y*	Periorbital	endocanthal height	0.089	0.303	0.906
*PHL_protrusion*	Perioral	philtrum protrusion	0.098	0.263	0.830
*DSM_length*	Nasal	dorsum length	0.534	0.383	0.818
*EXC_x*	Periorbital	exocanthal placement	0.582	0.291	0.807

**Table 5:** Attribute comparison of 40 female versus 40 male (pooled geographic ancestry). Biological sexes were found to differ in mean shape based on Wilks’s Lambda. Those individual attributes that were significantly different based on univariate t-tests with Bonferroni correction are listed. Attributes are sorted in order of decreasing difference between means (Δ) as quantified in terms of standard deviations.

Attribute	Region	Description	Female *X*	Male *X*	Δ
*GNA_length*	Lower Face	gnathion length	0.334	0.547	1.109
*CH_width*	Perioral	cheilion width	0.553	0.722	0.979
*GON_height*	Lower Face	gonion height	0.777	0.471	0.954
*TRG_x*	Mid Face	tragion placement	0.381	0.517	0.949
*SPC_weight*	Periorbital	suprapalpebral convexity	0.540	0.792	0.940
*TCH_protrusion*	Forehead	trichion protrusion	0.462	0.619	0.934
*SOR_protrusion*	Forehead	supraorbital ridge protrusion	0.246	0.446	0.930
*CHK_protrusion*	Mid Face	cheek protrusion	0.339	0.232	0.849
*PHL_length*	Perioral	philtrum length	0.412	0.569	0.825
*TRG_z*	Mid Face	tragion placement	0.712	0.453	0.746
*FOR_width*	Forehead	forehead width	0.659	0.810	0.745
*PHL_width*	Perioral	philtrum width	0.507	0.718	0.740

Size was found to be an important component of facial shape in the TFM analyses, but not the Procrustes. Multivariate analysis of covariance on the TFM data demonstrated that *meanVertexRadius* was significantly related to facial shape (*p* = 0.0005), while the Procrustes data showed no relationship between shape and the natural log of centroid size (*p* = 0.07753).

We also calculated the angles between vectors of shape difference for geographic ancestry group, sex, and size, represented by *meanVertexRadius* for the TFM data and the natural log of centroid size for the Procrustes data (Table 7). For the TFM, ancestry and sex are approximately orthogonal, a fact corroborated by the pattern of their scores for PC1 and PC2, while these two variables are only modestly correlated in the Procrustes data. Size and sex are also closely correlated in the TFM data. In the Procrustes data, geographic ancestry groups and size show only a weak relationship, whereas the correlation is stronger in the TFM data.

#### 4.2.3 Visualization of Procrustes data

**Fig 9:**
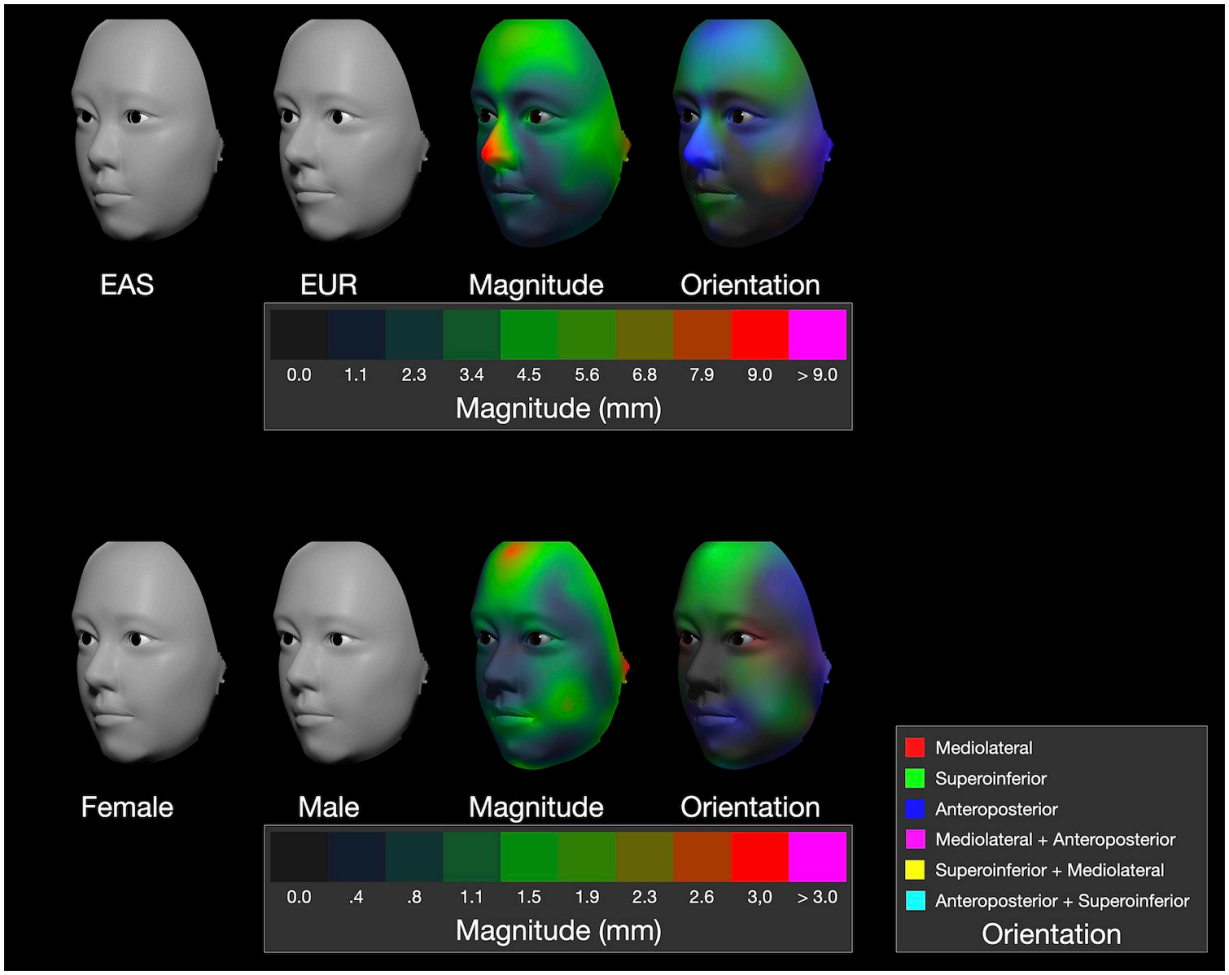
Visualization of four warps of the Procrustes data. The two geographic ancestry groups are shown in the upper row and the two sexes in the lower row. The magnitude heatmaps have different scales (red corresponds to 9 mm for ancestry differences and 3 mm for sex differences). The orientation of displacement is encoded in the ternary maps with the same convention as used elsewhere in this study.

The results for the Procrustes study are shown in Fig 9. Following the conventional procedure to visualize such data, a common polygonal mesh was ‘warped’ to match the average landmark positions for each of the four groups (the two ancestry groups and the two sexes). A polygonal mesh was derived from the TFM data by creating the grand mean for all 71 attribute variables across the 80 models (see the rightmost model in Fig 7B) then converting that model to a polygonal mesh. That polygonal mesh was landmarked (by SF) at the 43 positions in Fig 6 using Landmark Editor. Displacements for the 43 landmarks calculated for ancestry group, sex and size were then used to warp the landmarked mesh in Landmark editor. The differences in shape between warps was visualized by measuring the displacements at 2,096 homologous points and presenting their magnitude as a heatmap and their orientation of displacement as a ternary map (Fig 9).

The differences between the ancestry group warps (upper row in Fig 9) are primarily in the nose and forehead as revealed by the magnitude map. The ternary map shows that the displacements of the nose and forehead have both superoinferior (green) and anteroposterior (blue) components. To a lesser extent, there is also mediolateral (red) displacement in the vicinity of the cheeks. The difference in sex (lower row in Fig 9), while similar to those of ancestry as just mentioned, there is greater superoinferior displacement in the forehead, mediolateral displacement of the eyes, however less difference in the nose.

#### 4.2.4 Visualization of TFM data

**Fig 10:**
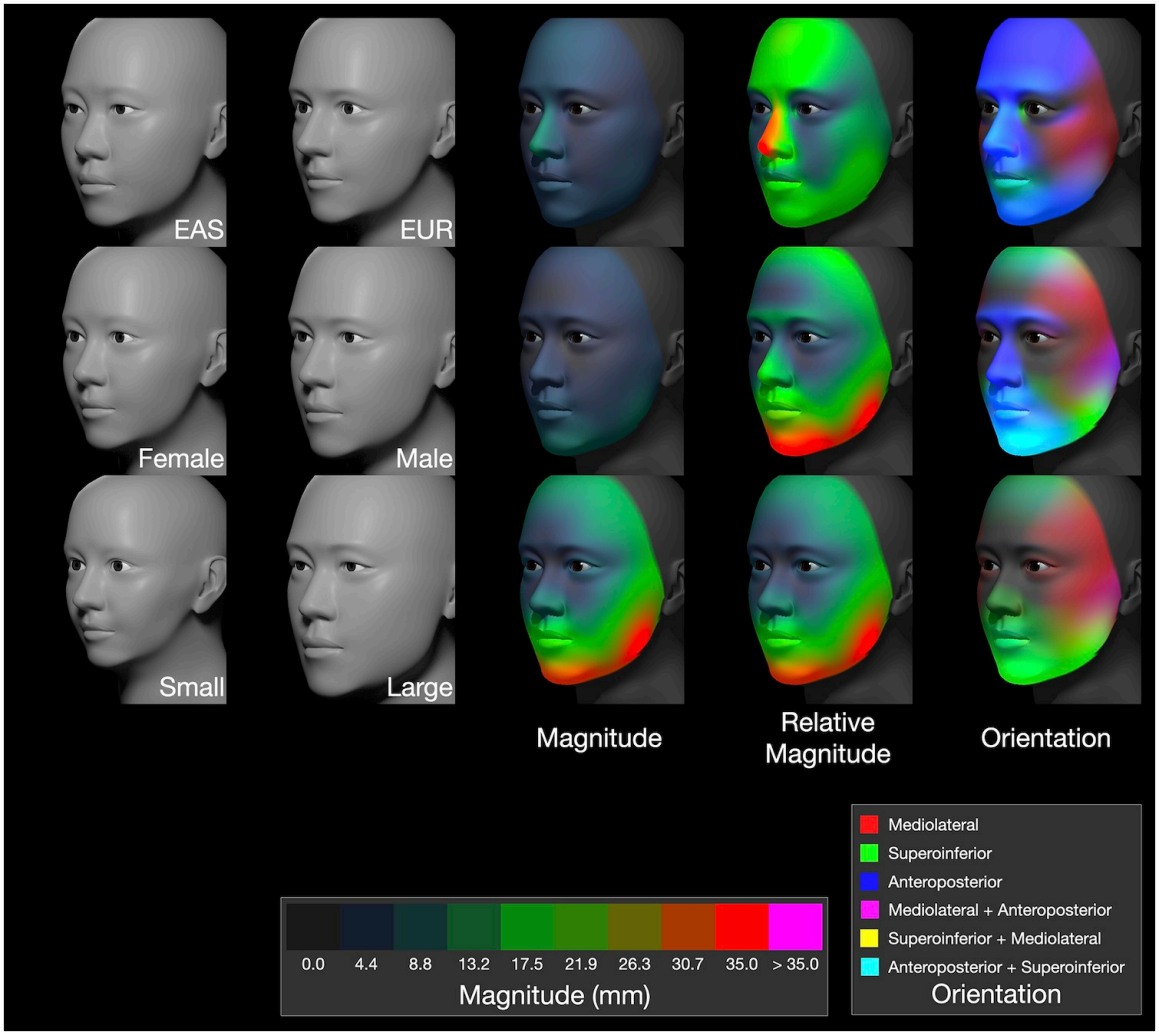
Visualization of differences based on the TFM dataset. Mean differences are shown for geographic ancestry group (top row), sex (middle row), and size (bottom row) based on the TFM dataset. The first two columns show the mean face associated with each factor. The third column shows the magnitude of difference with the same heatmap scale, where red corresponds to 35 mm (see key). Since differences in ancestry and sex are more subtle than the size differences, the fourth column shows their relative magnitudes scaled to the maximum displacement for each case (10.8, 9.7, and 35.0 mm for ancestry, sex, and size, respectively). The last column shows displacement orientation.

The results for the TFM study are shown in Fig 10. The models for EAS, EUR, and the two sexes are each based on averaged attribute values across 40 individuals; the ‘Small’ and ‘Large’ models were calculated by first creating a vector of coefficients from regression of TFM attribute values on *meanVertexRadius*. This vector was then scaled to represent the extremes of *meanVertexRadius* by subtracting the mean size of the smallest individual from the largest and dividing this number in half, resulting in a value of 10.575. The mean size vector was then multiplied by this value and added and subtracted from the grand mean TFM configuration. The third column shows magnitude heatmaps for each pair of averaged models, all with the same scale (where red indicates a displacement of 35 mm; see key). The fourth column shows heatmaps that are individually scaled to the maximum displacement in each pairwise comparison. The rightmost column shows the orientation of displacement.

The second row in Fig 10 shows the averaged models for the two sexes. Female faces are approximately 6.3% smaller in *meanVertexRadius* (81.7±2.7 mm versus 86.9±2.9 mm). While male supraorbital ridges are more pronounced, female foreheads have greater absolute protrusion. In the midface, the female cheeks protrude more, while the male face shows greater maxillary protrusion and a more robust mandible. These differences are tabulated in Table 6, where 18 attributes were found to be statistically significantly different.

**Table 6:** Attributes most strongly correlated with size as measured by *meanVertexRadius*. Size was found to have a significant relationship with shape based on Wilks’s Lambda. Those individual attributes that were significantly correlated with size with Bonferroni correction are listed. Attributes are sorted in order of decreasing correlation coefficient (*r*^*2*^).

Attribute	Region	Description	Slope	σ	Slope / σ	*p*	*r* ^*2*^
*TRG_x*	Mid Face	tragion placement x	0.027	0.143	0.191	2.01E-14	0.530
*GNA_length*	Lower Face	gnathion length	0.034	0.192	0.179	2.52E-12	0.469
*IPD_x*	Periorbital	interpupillary distance	0.025	0.166	0.153	1.34E-08	0.341
*CHK_width*	Mid Face	cheek width	0.026	0.177	0.150	3.18E-08	0.326
*ALA_width*	Nasal	alar width	0.020	0.140	0.143	1.44E-07	0.300
*TRG_z*	Mid Face	tragion placement z	-0.050	0.347	-0.143	1.73E-07	0.297
*GON_height*	Lower Face	gonion height	-0.043	0.320	-0.133	1.53E-06	0.258
*SPC_weight*	Periorbital	superior palpebral convexity	0.035	0.268	0.131	2.52E-06	0.249
*GON_width*	Lower Face	gonion width	0.015	0.119	0.128	4.29E-06	0.239
*ZYG_width*	Mid Face	zygion width	0.028	0.227	0.122	1.41E-05	0.216
*FOR_width*	Forehead	forehead width	0.025	0.202	0.122	1.45E-05	0.215
*MDB_width*	Lower Face	mandible width	0.027	0.234	0.118	2.99E-05	0.201
*PHL_length*	Perioral	philtrum length	0.022	0.190	0.115	4.94E-05	0.191
*DSM_length*	Nasal	dorsum length	0.020	0.173	0.113	6.13E-05	0.187
*GON_protrusion*	Lower Face	gonion protrusion	-0.030	0.280	0.108	1.34E-04	0.172
*SL_protrusion*	Perioral	superior labium protrusion	0.017	0.162	0.108	1.55E-04	0.169
*IL_protrusion*	Perioral	inferior labium protrusion	0.023	0.217	0.107	1.72E-04	0.166
*CH_width*	Perioral	cheilion width	0.018	0.173	0.104	2.54E-04	0.159
*TCH_protrusion*	Forehead	trichion protrusion	0.017	0.168	0.101	3.83E-04	0.150

Finally, the bottom row of Fig 10 shows the Small and Large models and the spatial pattern of their differences. The local deformations are similar to those for the sexes, except for the displacement of the supraorbital and nasal ridges. The absolute differences in the size (between Small and Large) are greater than those of sexual dimorphism. There is a greater anteroposterior component in the sexual dimorphism and a greater superoinferior component in the size differences.

**Fig 11:**
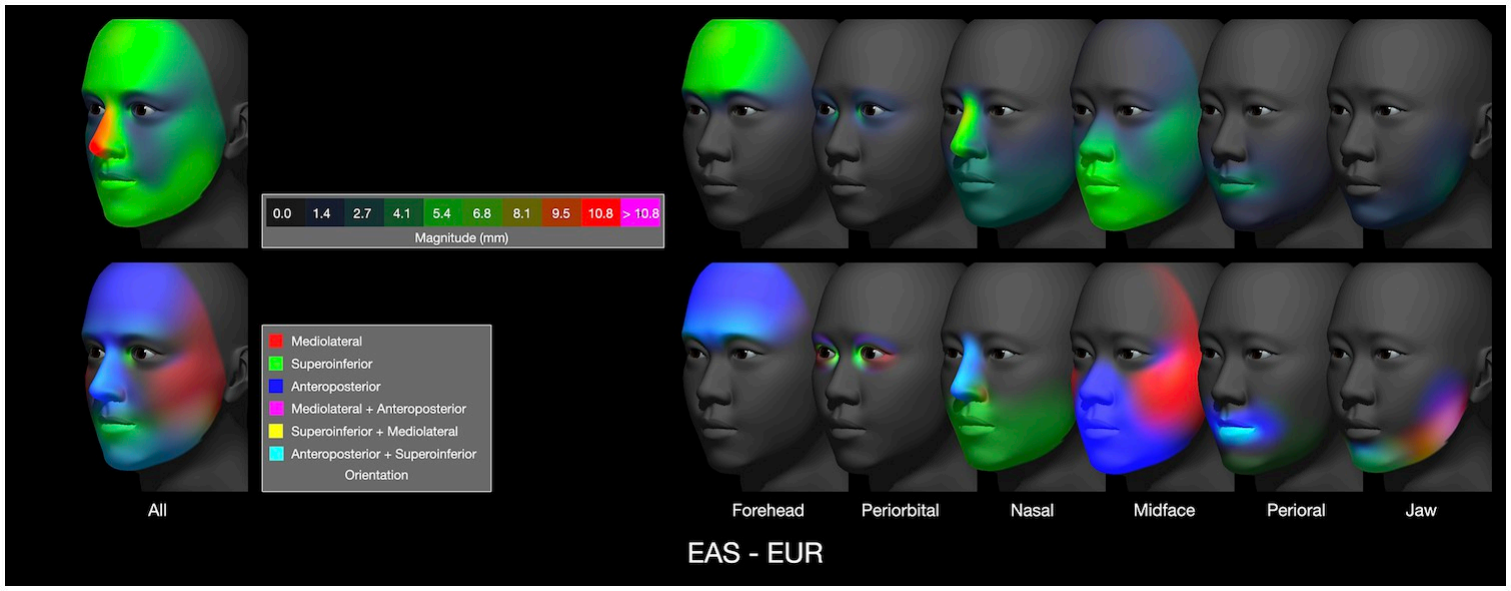
Differences between EAS and EUR on a per-region basis. Displacement magnitude is shown in the upper set of heatmaps, and orientation is shown in the lower set of ternary maps. The magnitude and orientation maps labeled ‘All’ are from the top row of Fig 10.

**Fig 12:**
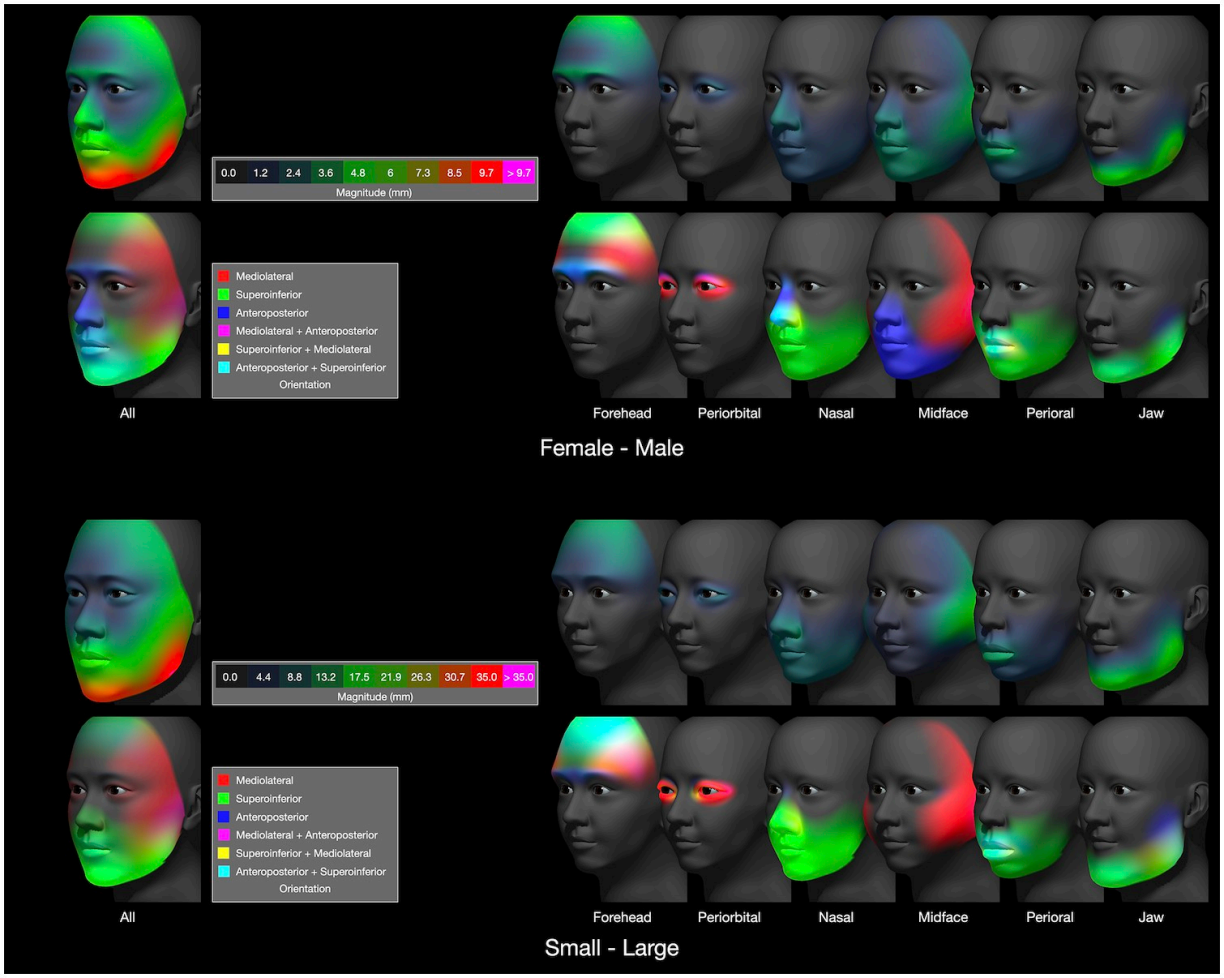
Visualization of sexual dimorphism (upper) and size (lower) on a per-region basis. Displacement magnitude is shown in the upper set of heatmaps, and orientation is shown in the lower set of ternary maps. The magnitude and orientation maps labeled ‘All’ are from the second and third rows of Fig 10.

Since our attributes are additive, we can isolate their specific contributions to overall shape by controlling which attributes are enabled in the model, either on a per-region basis or individually by attribute. For example, the differences associated with geographic ancestry (from the top row of Fig 10) are factored into contributions on a per-region basis (Fig 11). The greatest overall displacement is shown in red along the nasal ridge (with both anteroposterior and superoinferior components, as indicated by teal). The nasal region, in isolation, shows again a teal orientation which indicates differences in both nose protrusion and length, and the midface region, in isolation, reveals further protrusion (blue). Similar breakdowns are illustrated for sexual dimorphism and size in Fig 12.

### 4.3 Summary of results

Our topographical facial model was accurate at estimating both individual and averaged faces to within approximately two millimeters or better for 95% of the surface, and 82% within one millimeter (Table 2). These deviations are smaller than the average standard deviations for facial linear measurements for either sex within the human samples measured by Farkas et al. [21], both absolutely and relatively. Our results are comparable with those reported for the accuracy of localizing facial landmarks [84]. In contrast to landmarking a specific location, modeling involves adjusting multiple attributes to fit the morphology of a physical feature even where landmarks are sparse or nonexistent, such as across the smooth surface of the cheek or forehead. The measurement error is relatively small compared to the differences between most individuals and the statistical variation within our dataset and among our subgroups.

In our analysis of the 71 attributes from the TFM data we successfully distinguished ancestry and sex groups, using both MANOVA (Section 4.2.2) and PCA (Fig 8). Additionally, ancestry and sex were found to be largely independent (Table 7). In comparison, the analysis of the 43 Procrustes aligned landmarks also distinguished these groups based on MANOVA, but ancestry and sex were not found to be independent, but rather, partially correlated (Table 7). Additionally, PCA of the Procrustes data successfully separated ancestry groups along PC1, although not as completely as in the PCA of the TFM attribute data. The Procrustes PC1 also partially separated the sexes within each group, as did PC3 (Fig 8), whereas the PCA of TFM data concentrated sex differences along PC2 alone.

**Table 7:** Angles between group and size vectors. Angles near 90° or 270° degrees indicate statistical independence, while 0° or 180° indicate perfect correlation, values between these indicate degrees of correlation. Values above the diagonal are using TFM data, whereas those below are from Procrustes data. ‘Anc’ = geographic ancestry, ‘Sex’ = biological sex, ‘MVR’ = *meanVertexRadius*, and ‘CS’ = centroid size.

	**Anc**	**Sex**	**MVR**
**Anc**	—	92.9°	119.9°
**Sex**	54.5°	—	34.7°
**CS**	107.2°	61.5°	—

While both Procrustes and TFM analyses separated the different groups similarly, the TFM data showed a clear relationship with size, while the Procrustes data did not. Generalized Procrustes Analysis rescales each landmark configuration to unit centroid size, and then sequesters centroid size for each specimen as a new variable. Scale may be restored to a landmark configuration straightforwardly by multiplying each coordinate by centroid size. Given that the scan data were not normalized for size prior to modeling, the TFM attributes encode ‘form’ as distinct to ‘shape’ (*sensu* 79]), consistent with the recommendation that the decision on whether scale is left in the data depends upon the questions being asked [34]. In order to factor out size and/or orientation in TFM modeling it would be necessary to first normalize all sample scans prior to modeling.

## 5. Discussion

Comparing faces requires capturing homologous data. Conventional geometric morphometric methods use pointwise homologies. Anthropometric landmarks, however, have proven either too sparse or lacking in repeatability for some applications, and have led to techniques based upon increasingly-many point samples across the surface (*e*.*g*., [34, 64, 85, 86]). Another robust homology is provided by a set of topographical features (*e*.*g*., supraorbital *ridge*, labiomental *sulcus*, supra-alar *crease*) that reflect the common underlying structure of cranial soft tissues and osteology. To measure faces based upon topography rather than points, we first model the facial surface parametrically, then use those parameter values as surface measurements.

We derived a set of modeling parameters based upon how anatomical features are conventionally described. Anatomical description [4, 5, 6, 37–40], presumes that: 1) all faces share a common topography of distinct anatomical features, 2) each feature has an expected shape, 3) variations in that shape can be described by a few characteristics, 4) the variation in each characteristic is bracketed by pairs of adjectives (*e*.*g*., ‘broad’ versus ‘narrow’, ‘protruding’ versus ‘receded’), and 5) those characteristics are aligned with the anatomical planes and directions. That anatomical descriptive approach is intuitive, understandable, and expresses salient aspects of facial variation, but relies greatly on an implicit model of facial topography and morphology and expectations for ‘normal’ morphology. We adopt only a few aspects of this model, namely (1, 3, and 5). Regarding (2), our approach is not norm-based, and regarding (4), we derive continuous-valued variables (our ‘attributes’) from pairs of discrete adjectives, which are orthogonal given (5). Each attribute varies over a normalized (0.0 to 1.0) range, and serves as an independent parameter. The ‘meaning’ of an attribute is a three-dimensional shape, as Fig 2 demonstrated for the height and width attributes of a ridge.

The common topography of the human face is represented in our model by a deformable surface (specifically a subdivision surface), and each attribute is implemented as a local deformation of that surface (specifically a blendshape deformer). All feature attributes are local and their consequence on global shape is additive. This linear additivity is achieved by the displacement (translation) of control vertices, and their orthogonality is ensured by restricting the translations for each attribute to one of three orthogonal directions.

Parametric face modeling applications that are used for digital character design [60, 61, 87] often provide parameters that simultaneously control multiple local characteristics to manipulate global characteristics such as masculinity/femininity, ancestry, symmetry, age, and so forth. In the current study, however, a face model is created as a means to measure a face, hence each parameter provides a one-to-one linear relationship between attribute value and a corresponding translation deformation. This study identified 36 topographic features, for a total of 71 attributes, which were sufficient to approximate the facial surface of 80 individuals with accuracy mostly within 1 mm (Fig 7 and S7 Fig, Table 2), and identified areas where additional features could have improved the fit (such as the nasolabial fold).

### 5.1 Comparison of parametric and non-parametric approaches

In this section we examine some of the properties of our topographic modeling approach by comparing it with our conventional landmark-based Procrustes analysis of the same dataset.

#### 5.1.1 Information density

One clear difference is the granularity by which shape is encoded. A parametric model that incorporates a set of homologous topographic constraints can encode variations in that topography by a few variables, which would otherwise require a far greater number of point measurements. This efficiency comes at the expense of generality, however.

Both approaches can be regarded as deriving a vector of individual measurements. These two approaches differ dramatically, however, in terms of the information density of those measurements. The face is a useful place to compare these approaches, because, as noted previously, some facial areas are topographically simple and nearly devoid of landmarks [35, 64, 76]. In the absence of landmarks in those areas, GMM studies would require a high density of semilandmarks, particularly where the topography is complex, in order to ensure that sufficient morphology has been captured, resulting in a very large number of coordinate variables (*e*.*g*., [34]).

The TFM approach is successful at modeling human faces for two interrelated reasons. The constrained nature of human facial morphology allows us to focus on how faces vary. By capturing the shared topography in the model, we are left with only describing the specific morphological variation in each topographic feature across faces. By adopting conventional anatomical facial features, this decomposition of local morphology into attributes is consistent with those practices and results in each parameter providing an ‘information-dense’ measurement of facial shape.

#### 5.1.2 Form and shape

The differences in the way absolute scale is accommodated between Procrustes analysis and TFM is well illustrated by their seemingly contrasting results around sexual dimorphism and allometry. This is in part due to the TFM working in absolute scale, whereas GPA normalizes the data for size [28]. Conceptually, sexual dimorphism in facial shape has three potential contributions: absolute scale plus two types of shape difference, allometric and non-allometric [33]. For the TFM data, all three of these components apply, and the effects of absolute scale and allometry were confounded. Aspects of sexual dimorphism not related to size differences (absolute scale plus allometry), therefore, could be detected by comparing differences in shape attributable to sex and size (Fig 10). For the Procrustes data, differences in absolute scale are normalized during GPA, and as no significant allometric effect was detected; the differences between the sexes may be attributed to the non-allometric aspects of shape [23].

In the TFM data, sex and size have similar effects on shape (compare shared attributes in Tables 5 and 6; second and third rows in Fig 10). This was as expected given the sexes differed in size and there was a clear size effect on shape that likely includes both absolute scale and allometry. In the Procrustes data, however, the effects of size and sex on shape are not correlated. Absolute scale has been removed by GPA and no significant allometric effect was found in the Procrustes data.

The relationship between size and sex in the TFM data (Fig 10, third column) reflects the fact that the total range of size difference is approximately 4 times larger than the difference in size between the sexes. This hypothesis is supported by the low angle between the size and sex vectors (Table 5). In other words, there is a greater range of absolute facial size variation than can be attributed either to sex or ancestry. As observed in conventional anthropometry generally, there is often more variation within than across groups [21].

Nonetheless, there are subtle differences between sex and size factors in regions affected such as the supraorbital ridge, nasal ridge, and mouth (Fig 10). This indicates that there is a non-allometric component to human facial sexual dimorphism. In other words, even in a sample of females and males of equal facial size, there would still be a difference in facial shape attributable to sex. Specifically, males would show more anterior displacement of the supraorbital region, nasal ridge, and lower face (Fig 10).

If a size-normalized workflow were deemed important for the analysis, we could have pre-normalized the surface scan data before estimating model parameter values. A GPA-like alignment could have also been used prior to modeling as opposed to the anatomical orientation used here.

#### 5.1.3 Parametric visualization

While heatmaps, lollipops, and other graphical techniques are conventionally used to visualize the results of Procrustes studies, it is also common to create ‘warps’ of an exemplar mesh in order to *directly* visualize shape results [28, 29, 32]. In Fig 9, for example, the warps for EAS and EUR (upper row) are accompanied by heatmaps that indicate where they differ. This draws attention to the nose and forehead, but to discern *shape* differences at those places requires further scrutiny. Parametric models likewise provide for either indirect graphical annotation (*e*.*g*., by heatmap) or direct observation to appreciate shape differences (Fig 10). But since each attribute contributes independently and additively toward the overall shape of a model (Section 2.2), the shape differences between two models can be appreciated locally—by region (Fig 11), facial feature, or an individual attribute of a given feature. This provides some advantage in isolating differences. But nonetheless, shape differences are often subtle, and the incremental contributions of many attributes towards global shape are often difficult to appreciate by direct observation. It is therefore a common practice to exaggerate those differences [32].

**Fig 13:**
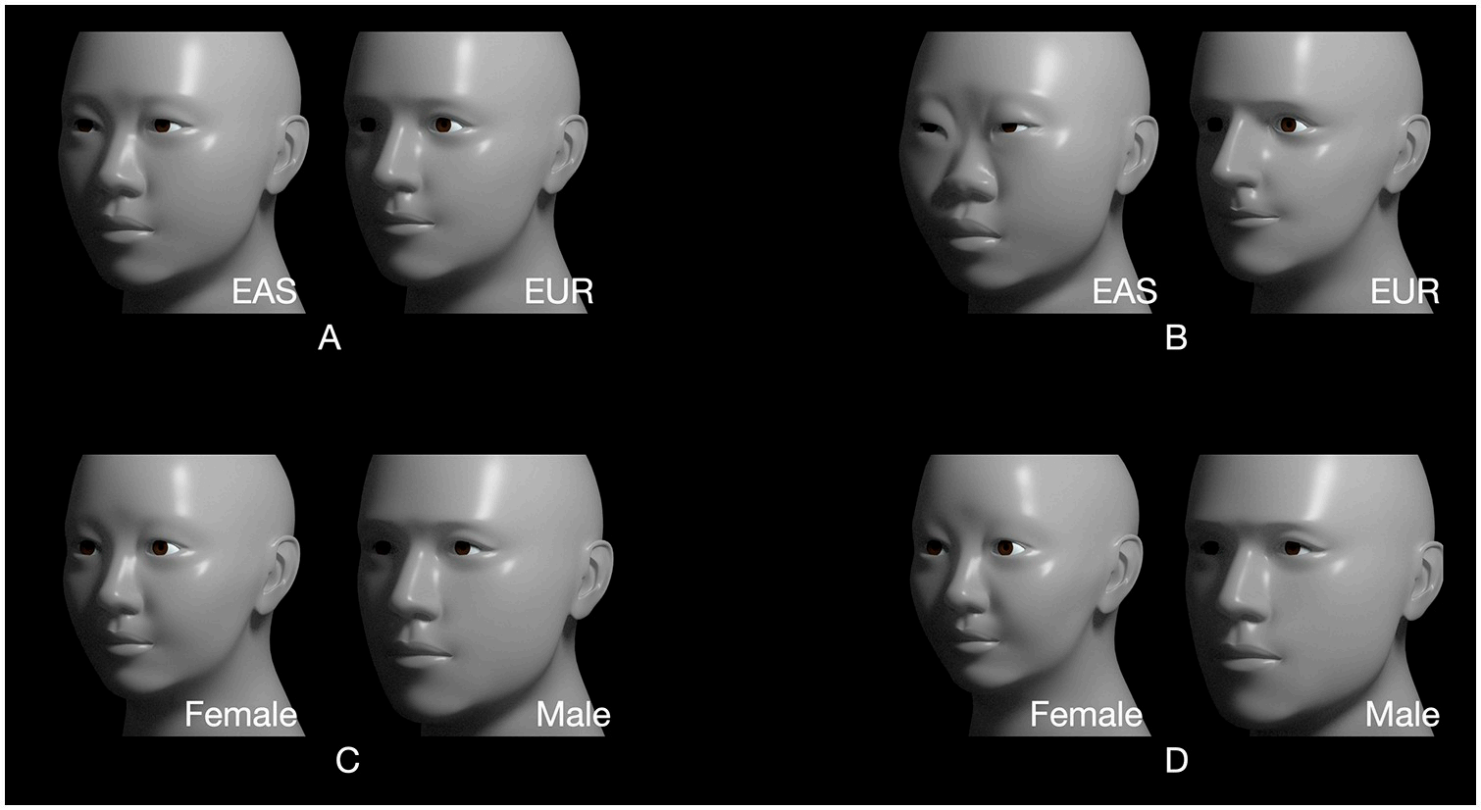
Exaggerating shape differences. The difference between two models can be exaggerated by increasing, for each of their 71 attributes, the difference between the value of that attribute relative to their mean by a multiplicative factor (2.0 in this demonstration). If, for example, a given attribute has values 0.3 and 0.5, they differ by ±0.1 relative to their mean, and with that difference doubled, the exaggerated values would be 0.2 and 0.6. Averaged models for EAS and EUR are shown in (A), each the average of 20 individual models (10 female and 10 male each). The corresponding exaggerated models are shown in (B). Likewise, (C) shows averaged models for females and males, each the average of 20 individual models (10 EAS and 10 EUR each), and their corresponding exaggerations are shown in (D). See also the corresponding animations S5 Movie (EAS-EUR) for (A), S6 Movie (exaggerations of EAS-EUR) for (B), S7 Movie (female-male) for (C), and S8 Movie (exaggerations of female-male) for (D).

Exaggeration is readily performed in the TFM (Fig 13). The pair of models in (A) are the computed averages for 40 EAS and 40 EUR, respectively. Note that each is a compilation of both sexes. The pair of models in (B) are exaggerations computed by amplifying the difference of each of the 71 attributes from the grand mean value by a given factor (in this case, by 2.0), creating a pair of caricatures of their features. Every aspect in which EAS differs from the mean of EAS and EUR is doubled, as are the differences in EUR from that mean. Likewise, the differences due to sexual dimorphism (Fig 13C) are shown exaggerated in Fig 13D (*cf*. [88]).

While geographic ancestry and sex are immediately recognizable in a glance (Fig 13A and 13C), appreciating *how* shape varies with ancestry or sex involves scrutiny—shifting visual attention alternately between the features in one image and their homologues in the other, seeking shape differences. Animation assists in this search. If one surface is observed to interpolate smoothly from one shape to the other, the surface will be seen to deform where the two shapes differ, creating visual motion that attracts attention [62, 89, 90]. Thus, animation draws our attention to where the shapes differ. In the TFM, animation is performed by linearly interpolating each attribute to ‘morph’ smoothly between two models. The efficacy of animation in visually revealing shape (and shape differences) can be compared with conventional scrutiny of static image pairs which present the same information (*cf*. S5–S8 Movies and the corresponding image pairs in Fig 13). While interpolation adds no new information, it visually reveals complex spatially-correlated patterns of shape difference that are often too subtle to be noticed from static images [89].

### 5.2 Applications

A central caveat of parametric modeling is the creation of a practical simplification of a family of objects where a general framework can be adjusted parametrically to approximate a range of specific instances. The parameters are of course specific to the given domain. This study explores the domain of facial modeling where the parameters were chosen to closely correspond to how faces are described. The model parameters serve both to measure a particular face and as a common basis for face description. Several applications beyond morphometrics are suggested; here we discuss two in particular: phenotyping and dysmorphologies.

Genome-wide association studies (GWAS) have been used in the search for the genetic basis of facial shape, where masses of genomic information are analyzed along with data on facial phenotypes (*e*.*g*., [91, 92]). In many such studies phenotypic data are typically selected *a priori*, *i*.*e*., a “phenotype-first” strategy [64]. For example, anthropometric distances (between landmarks) are often proposed as potential phenotypes [76, 93–101]. Alternatively, other studies have tried to avoid *a priori* selection of candidate phenotypes, by performing more data-intensive searches over a range of spatial scales [60, 92, 93] with the expectation that larger phenotypes may reflect developmental modules [102–105].

Ordered categorical scales are sometimes used to characterize facial traits that are less amenable to point-based morphometrics. For example, Adhikari et al. [94] defined a ‘nose profile’ trait with values ‘convex’, ‘straight’, or ‘concave’. In our approach, nose profile is not simplified to a single trait but rather the consequence of a combination of the protrusion of four topographic features along the ridge of the nose (*RAD_protrusion*, *DSM_protrusion*, *STB_weight*, and *TIP_protrusion*). This provides a more complete, continuous and objective quantification of facial features than can be described categorically, and with finer spatial localization. Our attributes also provide a more efficient characterization of facial shape, similar to the data-intensive approaches [64, 101, 102], but with far fewer variables. For example, the forehead and cheeks, which would require hundreds to thousands of vertices or semilandmarks to measure conventionally, can be modeled efficiently with a small set of attributes.

Clinical dysmorphology researchers set of characteristics [5, 6], and our parametric approach builds directly upon that literature. We offer the advantage of *quantifying* the specific traits that they describe categorically. While facial recognition and machine learning approaches are being proposed to automate the classification of various dysmorphologies, their proponents regard it “… unlikely that such software by itself will supersede the need for a good dysmorphology examination, even in the next 10–20 years” [106].

Therefore, our modeling approach has the potential to provide a quantitative, objective, and efficient characterization of facial phenotypes for GWAS analyses. Furthermore, because TFM data are aligned with conventional anatomical terminology and can be readily translated back to specimen space, any outcomes are readily visualized and described.

## Supporting information

S1 Fig Superoinferior and anteroposterior additivity. Varying *DSM_length* shifts the philtrum, mouth, and chin superoinferiorly relative to the model origin, while varying *PHL_length* shifts only the mouth and chin. Likewise, varying *MAX_protrusion* shifts the entire mid- and lower face anteroposteriorly relative to the origin. The overall protrusion of the tip of the nose is thus the sum of protrusion relative to the base, *TIP_protrusion*, and *MAX_protrusion*.
(TIF)

S2 Fig Mediolateral isolation. The medial features (those of the nose, philtrum, and mouth) and the lateral features (in the vicinity of the gonion, zygion, and tragion) are separated by the relatively featureless areas of the cheek and jaw. Varying the width of medial features (A versus B) does not shift mediolaterally the features of the side of the face, in contrast to superoinferior (S1 Fig A-D), and anteroposterior (S1 Fig E-H) attributes.
(TIF)

S3 Fig User interface. A composite scan of 20 EAS males in the process of being modeled with the TFM attributes of the nose region selected for adjustment. The modeling process involves progressively matching the shape of facial features proceeding from proximal to distal relative to the model origin (see Section 2.2).
(TIF)

S4 Fig Visualizing displacement magnitude. The conventional ‘jet’ heatmap (Mathworks Inc., Natick, MA, USA) is commonly used to visualize a distribution of scalar magnitudes across a surface, such as the disparity between a model and its corresponding digital scan (A). The various brilliant colors of the conventional jet map, however, distract from the intended goals of drawing attention to the regions of high magnitude. We therefore created a ‘dark jet’ heatmap (B), in which the chroma of the conventional ‘jet’ color spectrum is linearly desaturated towards gray for values at the low end of the range while at the high end of the range the colors converge upon those of the standard jet map. This draws attention away from low magnitude (gray) areas, and towards the high magnitude (colorful) areas. Moreover, the shading in the gray areas better conveys the three-dimensional shape of the underlying surface.
(TIF)

S5 Fig Visualizing displacement orientation. Three color channels are used to visualize the orientation of displacement between homologous points on two models, where red, green, and blue correspond to mediolateral, superoinferior, and anteroposterior, respectively. The color components are additive, *e*.*g*., blue-green indicates a combination of anteroposterior and superoinferior displacements (see key). Note that gray represents zero displacement.
(TIF)

S6 Fig Displacement orientation and spatial extent of the deformation associated with each attribute. Note how length attributes such as *DSM_length* and *PHL_length* shift distal features superoinferiorly and protrusion attributes such as *MAX_protrusion* shift distal features anteroposteriorly—see key in S5 Fig.
(TIF)

S7 Fig Evaluation of individual fit between model and scan. The individual models, each with its associated heatmap, showing the perpendicular separation between model and scan surfaces. The heatmap scale is 0.0 to 2.0 mm, as used in Fig 7.
(TIF)

S1 File Spreadsheet (CSV format) providing Procrustes coordinates and TFM attribute values for each of 80 models. (CSV)

S1 Movie Animation of 18 nasal attributes. (M4V)

S2 Movie Animation of 14 periorbital attributes. (M4V)

S3 Movie Examples of models created by the TFM. An animation shows continuous blends between 10 distinct models in succession, achieved by linearly interpolating all attributes simultaneously between each successive pair of models.
(M4V)

S4 Movie Animations of the 80 individual models. Each quadrant shows 20 models for the individuals in each group, with smooth transitions from one model to the next achieved by linear interpolation of model attributes.
(MOV)

S5 Movie Animation between the means of EAS and EUR. (MOV)

S6 Movie Animation between exaggerated means EAS and EUR. (MOV)

S7 Movie Animation of sexual dimorphism. (MOV)

S8 Movie Animation of exaggerated sexual dimorphism. (MOV)
