# Supplementary figures and images for: Facial modeling and measurement based upon homologous topographical features
Source: PLoS One. 2024 May 31;19(5):e0304561. doi: 10.1371/journal.pone.0304561 (PMC11142440; doi:10.1371/journal.pone.0304561)

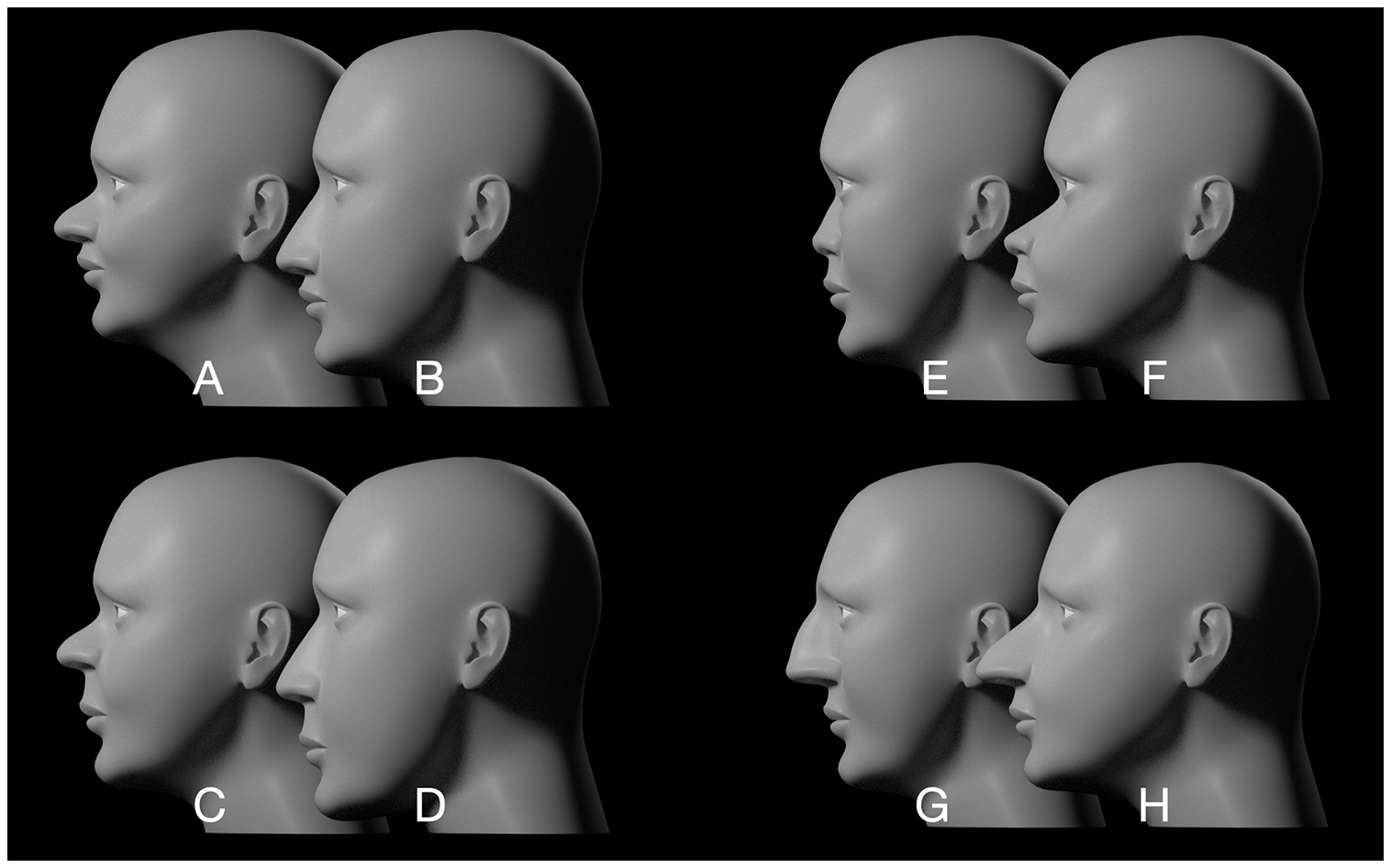

Supplement: S1 Fig — Varying DSM_length shifts the philtrum, mouth, and chin superoinferiorly relative to the model origin, while varying PHL_length shifts only the mouth and chin. Likewise, varying MAX_protrusion shifts the entire mid- and lower face anteroposteriorly relative to the origin. The overall protrusion of the tip of the nose is thus the sum of protrusion relative to the base, TIP_protrusion, and MAX_protrusion. (TIF) [file pone.0304561.s001.tif]

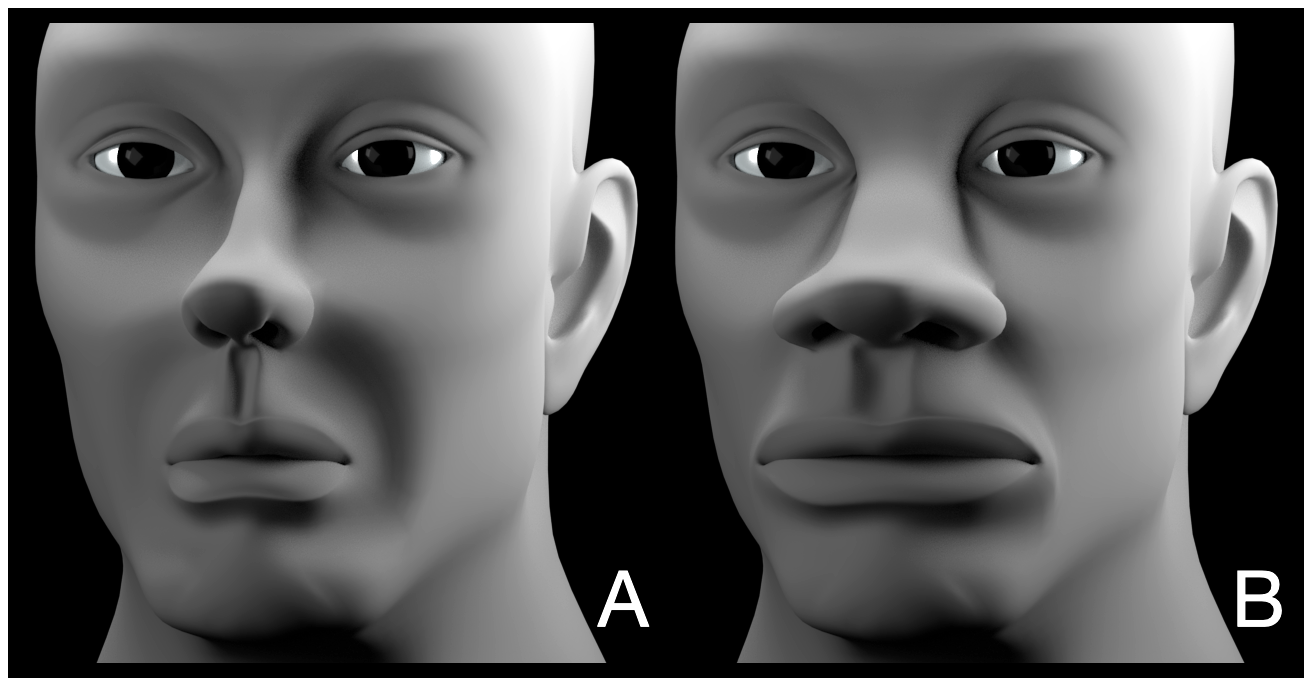

Supplement: S2 Fig — The medial features (those of the nose, philtrum, and mouth) and the lateral features (in the vicinity of the gonion, zygion, and tragion) are separated by the relatively featureless areas of the cheek and jaw. Varying the width of medial features (A versus B) does not shift mediolaterally the features of the side of the face, in contrast to superoinferior (S1 Fig A-D), and anteroposterior (S1 Fig E-H) attributes. (TIF) [file pone.0304561.s002.tif]

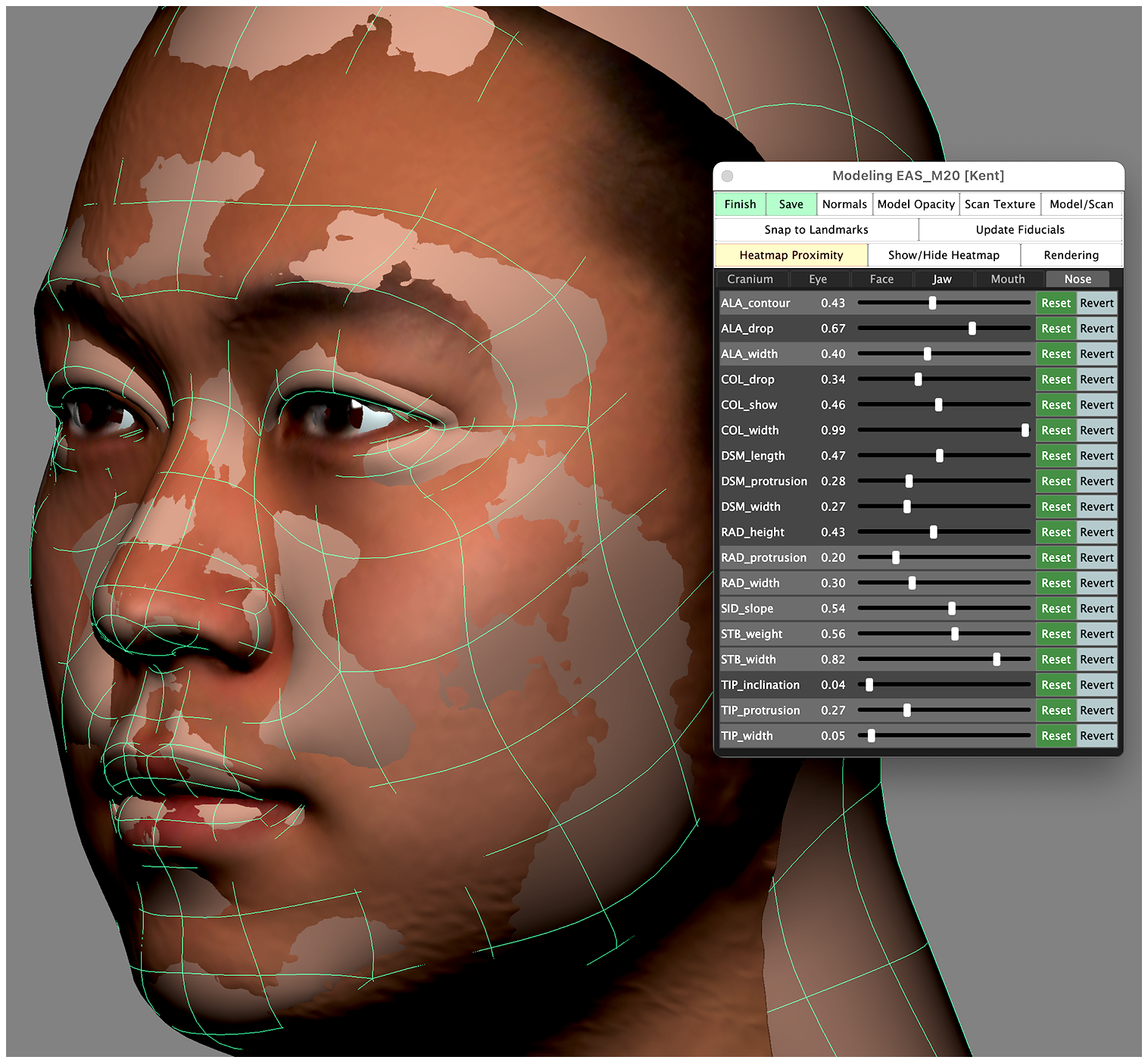

Supplement: S3 Fig — A composite scan of 20 EAS males in the process of being modeled with the TFM attributes of the nose region selected for adjustment. The modeling process involves progressively matching the shape of facial features proceeding from proximal to distal relative to the model origin (see Section 2.2). (TIF) [file pone.0304561.s003.tif]

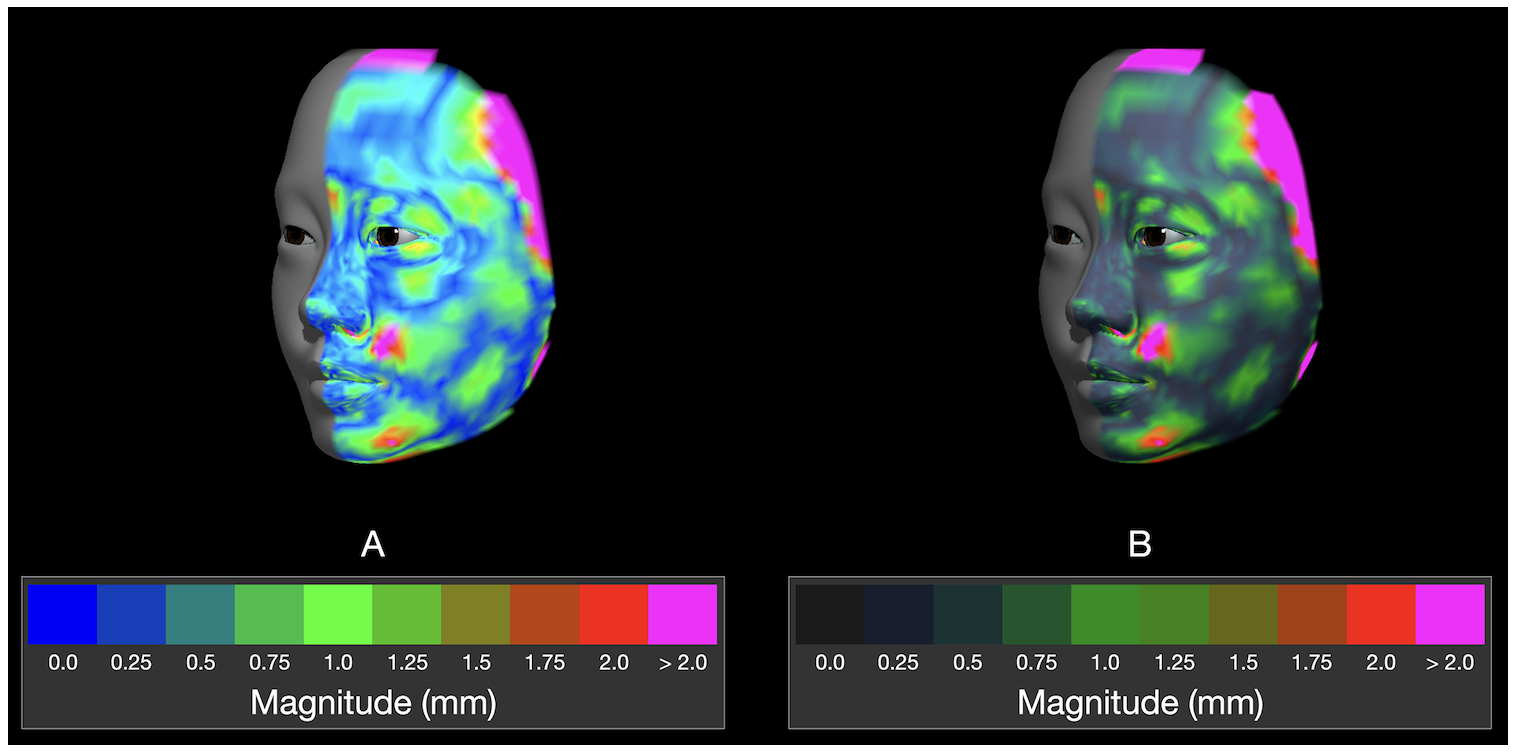

Supplement: S4 Fig — The conventional ‘jet’ heatmap (Mathworks Inc., Natick, MA, USA) is commonly used to visualize a distribution of scalar magnitudes across a surface, such as the disparity between a model and its corresponding digital scan (A). The various brilliant colors of the conventional jet map, however, distract from the intended goals of drawing attention to the regions of high magnitude. We therefore created a ‘dark jet’ heatmap (B), in which the chroma of the conventional ‘jet’ color spectrum is linearly desaturated towards gray for values at the low end of the range while at the high end of the range the colors converge upon those of the standard jet map. This draws attention away from low magnitude (gray) areas, and towards the high magnitude (colorful) areas. Moreover, the shading in the gray areas better conveys the three-dimensional shape of the underlying surface. (TIF) [file pone.0304561.s004.tif]

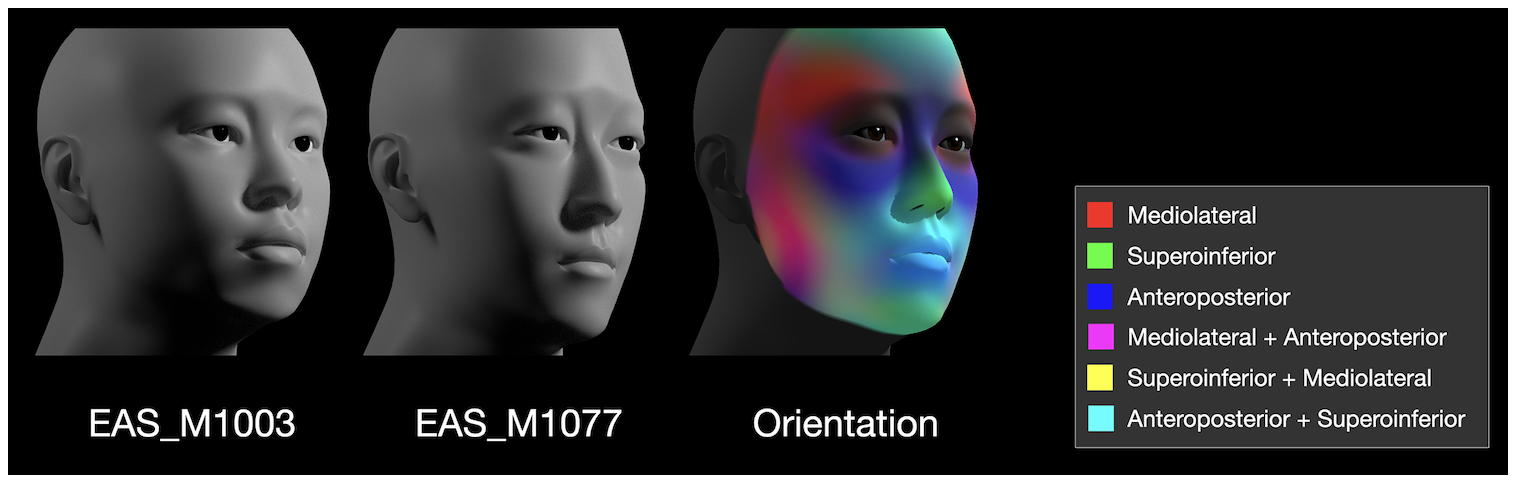

Supplement: S5 Fig — Three color channels are used to visualize the orientation of displacement between homologous points on two models, where red, green, and blue correspond to mediolateral, superoinferior, and anteroposterior, respectively. The color components are additive, e.g., blue-green indicates a combination of anteroposterior and superoinferior displacements (see key). Note that gray represents zero displacement. (TIF) [file pone.0304561.s005.tif]

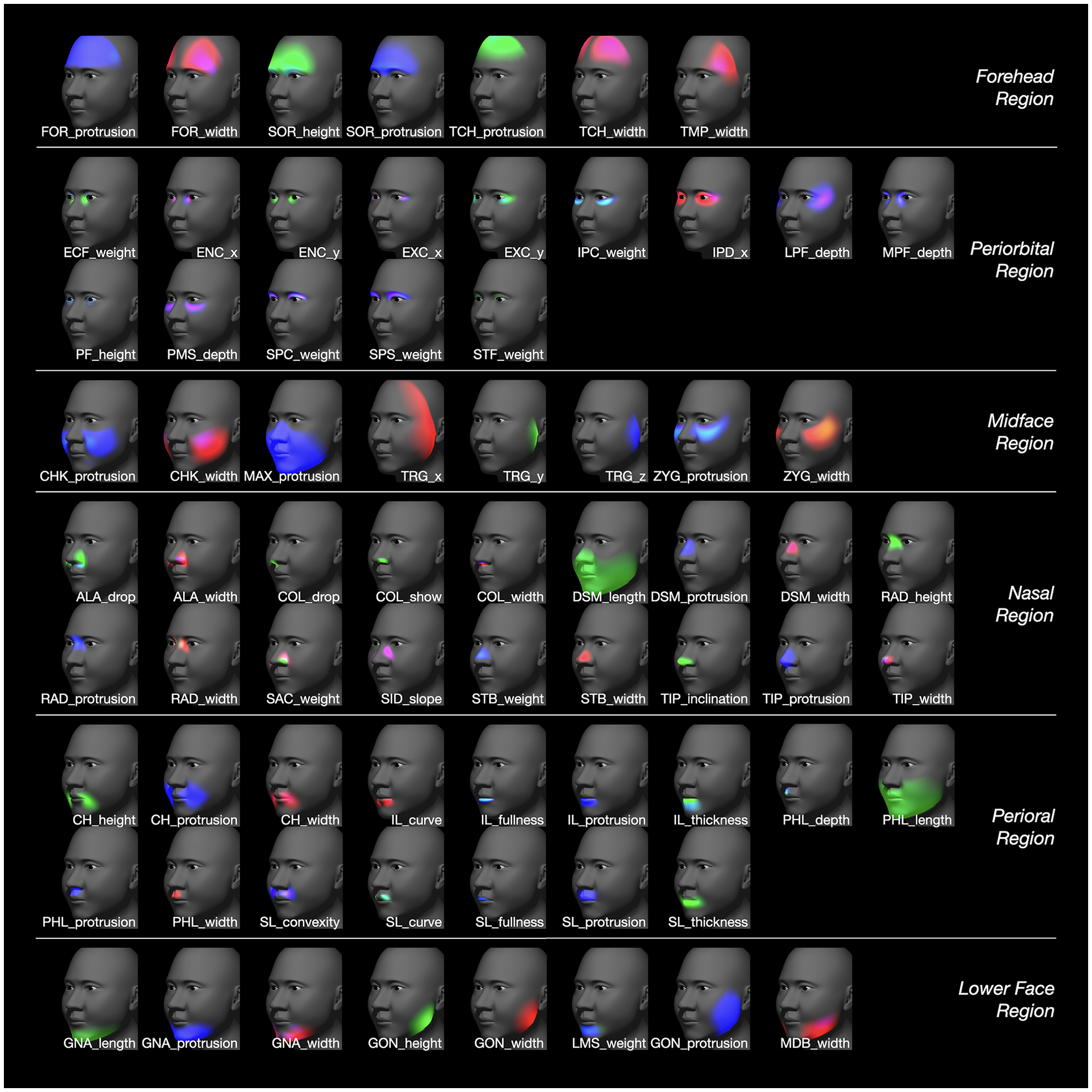

Supplement: S6 Fig — Note how length attributes such as DSM_length and PHL_length shift distal features superoinferiorly and protrusion attributes such as MAX_protrusion shift distal features anteroposteriorly—see key in S5 Fig. (TIF) [file pone.0304561.s006.tif]

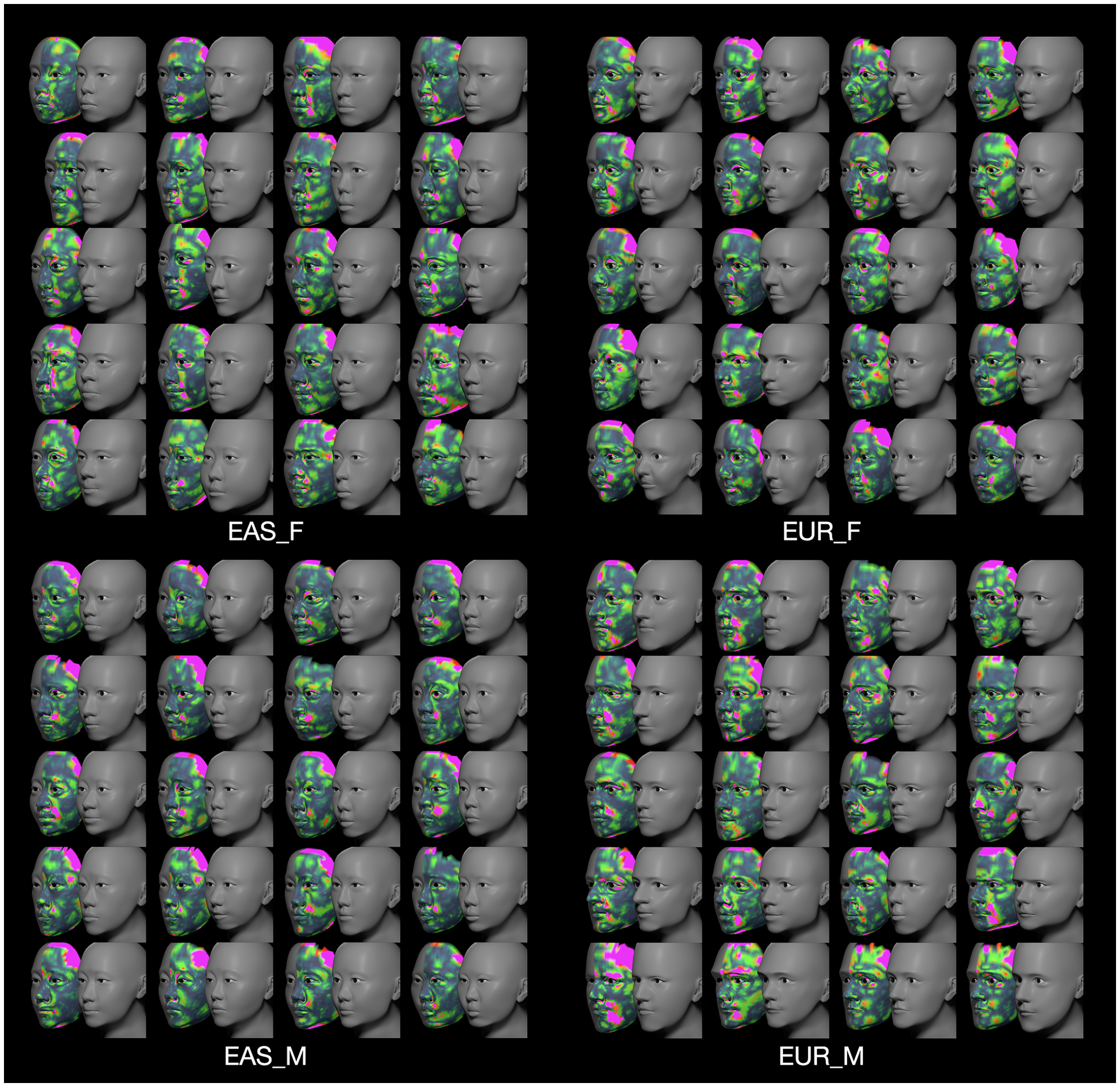

Supplement: S7 Fig — The individual models, each with its associated heatmap, showing the perpendicular separation between model and scan surfaces. The heatmap scale is 0.0 to 2.0 mm, as used in Fig 7. (TIF) [file pone.0304561.s007.tif]
